# Design, synthesis and evaluation of quinolinone derivatives containing dithiocarbamate moiety as multifunctional AChE inhibitors for the treatment of Alzheimer’s disease

**DOI:** 10.1080/14756366.2019.1687460

**Published:** 2019-11-07

**Authors:** Jie Fu, Fengqi Bao, Min Gu, Jing Liu, Zhipeng Zhang, Jiaoli Ding, Sai-Sai Xie, Jinsong Ding

**Affiliations:** a Xiangya School of Pharmaceutical Sciences, Central South University, Changsha, China; b Jiangsu Zeyun Pharmaceutical Co., Ltd, Xibei Town Industrial Park, Wuxi, China; c National Pharmaceutical Engineering Center for Solid Preparation in Chinese Herbal Medicine, Jiangxi University of Traditional Chinese Medicine, Nanchang, China; d School of Pharmacy, Jiangxi University of Traditional Chinese Medicine, Nanchang, PR China

**Keywords:** Dithiocarbamate, quinolinone, cholinesterase, Alzheimer’s disease, multifunctional inhibitors

## Abstract

A series of novel quinolinone derivatives bearing dithiocarbamate moiety were designed and synthesised as multifunctional AChE inhibitors for the treatment of AD. Most of these compounds exhibited strong and clearly selective inhibition to *ee*AChE. Among them, compound **4c** was identified as the most potent inhibitor to both *ee*AChE and *h*AChE (IC_50_ = 0.22 μM for eeAChE; IC_50_ = 0.16 μM for *h*AChE), and it was also the best inhibitor to AChE-induced Aβ aggregation (29.02% at 100 μM) and an efficient inhibitor to self-induced Aβ aggregation (30.67% at 25 μM). Kinetic and molecular modelling studies indicated that compound **4c** was a mixed-type inhibitor, which could interact simultaneously with the catalytic anionic site (CAS) and the peripheral anionic site (PAS) of AChE. In addition, **4c** had good ability to cross the BBB, showed no toxicity on SH-SY5Y neuroblastoma cells and was well tolerated in mice at doses up to 2500 mg/kg (po).

## 1. Introduction

Alzheimer’s disease (AD), a progressive and irreversible neurodegenerative disorder, is the most common cause leading to memory impairment and dementia in elderly people^1^. According to World Alzheimer Report, nearly 50 million people worldwide are living with dementia in 2018, and the number is likely to reach 152 million by 2050^2^. This disease has higher use and costs of health care and long-term care services than other conditions^3^. Therefore, such a large number of patients will bring heavy burden to public health system in the future.

AD was first identified >100 years ago, but the exact aetiology is still largely unknown. Many clinical hallmarks, including deficits of acetylcholine (ACh), amyloid-β (Aβ) peptide deposits, oxidative stress, dyshomeostasis of biometals and hyperphosphorylated tau protein, are thought to be associated with the initiation and development of AD. Over the past years, although great efforts have been devoted to develop novel drugs to treat AD by both pharmaceutical companies and academic institutions, no new drugs have entered the clinical market and >200 drug candidates ended up with failure in advanced clinical trials. Currently, the clinical treatment of AD is mainly based on the cholinergic hypothesis, which states that improving the ACh level in brain through inhibition of ACh hydrolysis can alleviate the memory loss and cognitive decline related to AD^4^^,^^5^. At the neuronal level, there are two types of cholinesterases (ChEs) that can catalyse ACh hydrolysis, namely acetylcholinesterase (AChE) and butyrylcholinesterase (BuChE)^6^. However, in contrast to AChE, BuChE is mainly distributed in the peripheral areas, and the inhibition of BuChE may cause side effect in the peripheral system^7^^,^^8^. Thus, selective inhibition of AChE seems more beneficial for AD treatment.

In addition to cholinergic hypothesis, another hypothesis, so-called amyloid-β (Aβ) pathological hypothesis, has attracted great interest in recent years^9^. This hypothesis posits that the gradual accumulation and aggregation of Aβ peptide in the brain is a central event that results in neurodegeneration and dementia^10^^,^^11^. Aβ peptides consist of 39–43 residues, which are generated via sequential scission of the amyloid precursor protein (APP) by β- and γ-secretases. Aβ peptides can aggregate into monomers, oligomers and large Aβ plaques, which initiate a neurodegenerative cascade that involves inflammation, oxidative stress, and mitochondrial dysfunction, eventually leading to neuronal death and cognitive dysfunction^12^. Therefore, prevention of Aβ aggregation provides another therapeutic approach to halt AD pathogenesis.

In fact, besides the vital role in nerve transmission, AChE also plays an important role in processing of Aβ peptides^13^. The crystallographic structure of AChE shows that it has a nearly 20 Å deep narrow gorge which consists of two binding sites: a catalytic active site (CAS) at the bottom of the gorge and a peripheral anionic site (PAS) near the entry of the gorge^14^. Generally, inhibitors that bind to either one site can inhibit the AChE. However, recent reports indicate that, apart from catalysing ACh hydrolysis, the PAS also has a close relationship with the Aβ aggregation. The PAS can accelerate the formation of Aβ aggregation and amyloid fibril. The interaction between Aβ and the PAS produces stable AChE-Aβ complexes, which are more toxic than Aβ peptide aggregates^15^^,^^16^. Accordingly, dual binding site inhibitors able to interact with both the CAS and PAS will be more promising for treating AD, as they can not only improve cognition by inhibiting AChE, but also provide additional benefit through reducing the Aβ aggregation induced by AChE. The basic precept of designing dual binding site AChE inhibitors is to take three parts to fulfil the structural requirements of AChE. The three parts are a PAS binding moiety, a CAS binding moiety and a linker connecting these two moieties.

To date, there are four AChE inhibitors and one N-methyl-D-aspartate receptor antagonist that have been approved for the treatment of AD in clinic. However, due to the complex aetiology of AD, these single-target drugs can only achieve some temporary amelioration of the symptoms instead of slowing or halting the disease progress. Thus, a more appropriate approach for treating this disease is development of multifunctional molecules that can simultaneously modulate multiple targets or pathways related to AD. The most popular method for obtaining these molecules is to design a dual binding site AChE inhibitor and make it exert additional activities because of the fragments introduced.

The quinolinone scaffold is prevalent in a variety of pharmacologically active synthetic and natural compounds^17^. The reported biological activities of quinolinone derivatives including antioxidation, anti-osteoporosis, anti-influenza and anticancer activities, etc.^18^. Recent studies indicated that several quinolinone derivatives also presented potent AChE inhibitory activity, and molecular docking studies showed that the quinolinone moiety could bind to the PAS through a π-π stacking interaction^19^^,^^20^. Therefore, it can serve as a PAS binding moiety for design of dual binding site inhibitor. On the other hand, dithiocarbamate is a versatile pharmacophore, which has been receiving intense attention in recent years. Very recently, our group found that this moiety could interact with CAS of AChE, and many compounds possessing this moiety also displayed promising inhibitory activity for self-induced Aβ aggregation^21^^,^^22^. Meanwhile, unlike the benzyl piperidine of donepezil or tacrine that have been widely used as CAS binding group for design of multifunctional AChE inhibitors, the dithiocarbamate is still rarely used. Therefore, it is interesting to explore this scaffold as a CAS binding group to design multifunctional AChE inhibitors.

**Figure 1.**
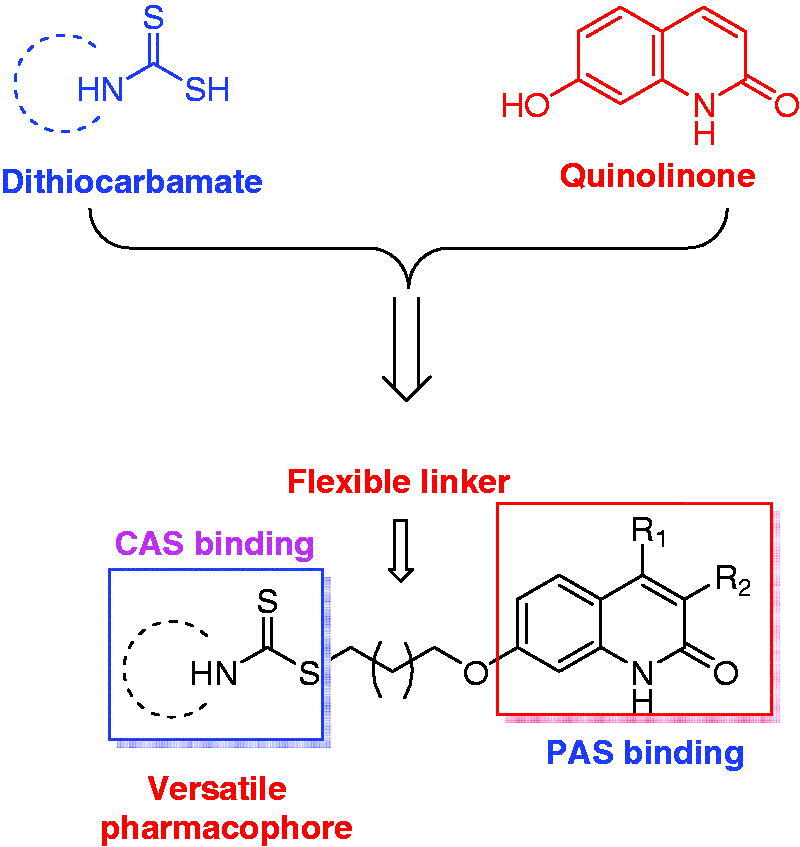
Rational design of quinolinone derivatives as multifunctional AChE inhibitors for the treatment of AD.

Based on the facts and considerations mentioned above, in present study, we attempted to connect quinolinone fragment with dithiocarbamate moiety to design a series of new compounds as multifunctional dual binding site AChE inhibitors for the treatment of AD (Figure 1). The quinolinone was selected to inhibit AChE-induced Aβ aggregation by binding to PAS, while the dithiocarbamate moiety was used to inhibit AChE through interacting with the CAS. A flexible linker was exploited to connect these two moieties, because such linker could allow designed compounds simultaneously bind to both CAS and PAS. In addition, since our previous studies indicated that compounds bearing dithiocarbamate moiety could efficiently inhibit self-induced Aβ aggregation, these new compounds were also expected to present similar activity, thereby exerting multifunctional effects. Furthermore, in our initial step, a piperidine dithiocarbamate moiety was first used as a starting fragment to explore the optimal linker length, because the previous studies suggested that this moiety could potently and selectively inhibit AChE. And once the optimal length was determined, various secondary amine groups were introduced to dithiocarbamate moiety for further structure-activity relationship (SAR) studies. All designed compounds were synthesised and evaluated for their ability to inhibit ChEs and cross the blood-brain barrier (BBB). The compounds with potential activities were also chosen for studies including the inhibitory evaluation of *h*AChE and Aβ aggregation, kinetic study of AChE inhibition, and molecular modelling studies of their binding modes with AChE and Aβ_1-42_. Finally, the most promising compound was selected to test its cytotoxicity in SH-SY5Y neuroblastoma cells and acute toxicity in mice. Herein, we report the design, synthesis and evaluation of a series of novel quinolinone derivatives containing dithiocarbamate moiety as multifunctional AChE inhibitors for AD treatment.

## 2. Experimental

### 2.1. Chemistry

All reagents and solvents were obtained from commercial suppliers and used without further purification unless specified. The reactions were monitored by analytical thin-layer chromatography on Silica Gel aluminium sheets and visualised with a UV lamp. Melting points were determined with an XT-4 micromelting point apparatus and are uncorrected. The NMR spectra were recorded on a Bruker ACF-600 spectrometer using the tetramethylsilane (TMS) as internal standard. High resolution mass spectra (HRMS) were taken on a Triple TOF 5600 spectrometer (AB Sciex).

### 2.2. General procedures for the preparation of compounds 3a-e and 8a-b

The intermediates **2** and 7**a-b** were prepared according to the literature methods^20^^,^^23^^,^^24^, and the structures of these compounds were characterised by mass spectra. Then, to a mixture of **2**/7**a-b** (5.0 mmol) and anhydrous K_2_CO_3_ (1.4 g, 10 mmol) in acetone (15 mL) was added appropriate α, ω-dibromoalkanes (50 mmol), and the reaction was refluxed under stirring for 4 h. After completing, the mixture was filtered, and the filtrate was evaporated under reduced pressure. The obtained residue was purified by silica gel chromatography with PE/EA (20:1) as eluent to give compounds **3a-e** and **8a-b** as white solid.

#### 2.2.1. 7–(2-bromoethoxy)quinolin-2(1H)-one (3a)

Yield 77%. ^1^H NMR (600 MHz, CDCl_3_) *δ* 7.77 (d, *J* = 9.3 Hz, 1H), 7.48 (d, *J* = 8.6 Hz, 1H), 6.91 (d, *J* = 2.4 Hz, 1H), 6.84 (dd, *J* = 8.6, 2.4 Hz, 1H), 6.59 (d, *J* = 9.3 Hz, 1H), 4.35 (t, *J* = 6.3 Hz, 2H), 3.69 (t, *J* = 6.3 Hz, 2H).

#### 2.2.2. 7–(3-bromopropoxy)quinolin-2(1H)-one (3 b)

Yield 79%. ^1^H NMR (600 MHz, CDCl_3_) *δ* 7.79 (d, *J* = 9.4 Hz, 1H), 7.50 (d, *J* = 8.7 Hz, 1H), 6.93 (d, *J* = 2.4 Hz, 1H), 6.86 (dd, *J* = 8.7, 2.4 Hz, 1H), 6.61 (d, *J* = 9.4 Hz, 1H), 4.24 (t, *J* = 5.8 Hz, 2H), 3.65 (t, *J* = 6.4 Hz, 2H), 2.39 (p, *J* = 6.1 Hz, 2H).

#### 2.2.3. 7–(4-bromobutoxy)quinolin-2(1H)-one (3c)

Yield 78%. ^1^H NMR (600 MHz, CDCl_3_) *δ* 7.76 (d, *J* = 9.3 Hz, 1H), 7.47 (d, *J* = 8.6 Hz, 1H), 6.86 (d, *J* = 2.3 Hz, 1H), 6.83 (dd, *J* = 8.6, 2.3 Hz, 1H), 6.58 (d, *J* = 9.4 Hz, 1H), 4.12 (t, *J* = 6.0 Hz, 2H), 3.53 (t, *J* = 6.6 Hz, 2H), 2.12 (p, *J* = 6.8 Hz, 2H), 2.04 – 1.99 (m, 2H).

#### 2.2.4. 7-((5-bromopentyl)oxy)quinolin-2(1H)-one (3d)

Yield 76%. ^1^H NMR (600 MHz, CDCl_3_) *δ* 7.76 (d, *J* = 9.4 Hz, 1H), 7.46 (d, *J* = 9.3 Hz, 1H), 6.87 (dd, *J* = 9.3, 2.4 Hz, 1H), 6.83 (d, *J* = 2.4 Hz, 1H), 6.56 (d, *J* = 9.4 Hz, 1H), 4.10 (t, *J* = 6.1 Hz, 2H), 3.45 (t, *J* = 6.1 Hz, 2H), 1.98 (br s, 2H), 1.87 (br s, 2H), 1.67 (br s, 2H).

#### 2.2.5. 7-((6-bromohexyl)oxy)quinolin-2(1H)-one (3e)

Yield 74%. ^1^H NMR (600 MHz, CDCl_3_) *δ* 7.76 (d, *J* = 9.4 Hz, 1H), 7.46 (d, *J* = 9.3 Hz, 1H), 6.89 – 6.72 (m, 2H), 6.56 (d, *J* = 9.4 Hz, 1H), 4.07 (t, *J* = 6.3 Hz, 2H), 3.44 (t, *J* = 6.8 Hz, 2H), 1.97 – 1.73 (m, 4H), 1.53 (p, *J* = 3.6 Hz, 4H).

#### 2.2.6. 7–(4-bromobutoxy)-4-methylquinolin-2(1H)-one (8a)

Yield 77%. ^1^H NMR (600 MHz, CDCl_3_) *δ* 7.59 (d, *J* = 8.7 Hz, 1H), 6.85 (br s, 1H), 6.83 (br s, 1H), 6.46 (s, 1H), 4.11 (t, *J* = 6.0 Hz, 2H), 3.51 (t, *J* = 6.5 Hz, 2H), 2.48 (s, 3H), 2.10 (p, *J* = 6.8 Hz, 2H), 1.99 (p, *J* = 6.8 Hz, 2H).

#### 2.2.7. 7–(4-bromobutoxy)-3,4-dimethylquinolin-2(1H)-one (8 b)

Yield 75%. ^1^H NMR (600 MHz, CDCl_3_) *δ* 7.63 (d, *J* = 9.0 Hz, 1H), 6.85 (dd, *J* = 9.0, 2.5 Hz, 1H), 6.70 (d, *J* = 2.5 Hz, 1H), 4.12 (t, *J* = 6.0 Hz, 2H), 3.53 (t, *J* = 6.5 Hz, 2H), 2.45 (s, 3H), 2.27 (s, 3H), 2.09 (p, *J* = 6.8 Hz, 2H), 1.98 (p, *J* = 6.8 Hz, 2H).

### 2.3. General procedure for the preparation of compounds 4a-m and 9a-b

To a solution of appropriate secondary amine (1.2 mmol) and TEA (131 mg, 1.3 mmol) in DMF (2 mL) was added CS_2_ (99 mg, 1.3 mmol) drop wise. After stirring for 5 min, another solution containing corresponding 2-quinolinone derivatives **3a-e** or **8a-b** (1.2 mmol) in DMF (3 mL) was added, and the mixture was stirred at room temperature for 12 h. After completing, the mixture was diluted with water, and the standard extraction procedure was carried out to afford the crude product, which was purified by silica gel chromatography with PE/EA (10:1) as eluent to obtain the target compounds.

#### 2.3.1. 2-((2-oxo-1,2-dihydroquinolin-7-yl)oxy)ethyl piperidine-1-carbodithioate (4a)

Yield 86%; mp 221–222 °C; ^1^H NMR (600 MHz, DMSO-*d*_6_) *δ* 11.56 (s, 1H), 7.81 (d, *J* = 9.5 Hz, 1H), 7.57 (d, *J* = 8.6 Hz, 1H), 6.83 (dd, *J* = 8.6, 2.4 Hz, 1H), 6.80 (d, *J* = 2.5 Hz, 1H), 6.31 (dd, *J* = 9.4, 1.1 Hz, 1H), 4.24 (t, *J* = 6.2 Hz, 4H), 3.92 (s, 2H), 3.71 (t, *J* = 6.3 Hz, 2H), 1.76 – 1.32 (m, 6H). ^13 ^C NMR (150 MHz, DMSO-*d*_6_) *δ* 193.33, 162.64, 160.30, 141.04, 140.44, 129.84, 119.24, 114.03, 111.07, 99.27, 66.68, 53.09, 51.49, 35.62, 26.29, 25.66, 23.99. HRMS: calcd for C_17_H_21_N_2_O_2_S_2_ [M + H]^+^ 349.1039, found 349.1028.

#### 2.3.2. 3-((2-oxo-1,2-dihydroquinolin-7-yl)oxy)propyl piperidine-1-carbodithioate (4 b)

Yield 83%; mp 186–187 °C; ^1^H NMR (600 MHz, CDCl_3_) *δ* 12.15 (s, 1H), 7.78 (d, *J* = 9.3 Hz, 1H), 7.48 (d, *J* = 8.6 Hz, 1H), 6.91 – 6.81 (m, 2H), 6.60 (d, *J* = 9.4 Hz, 1H), 4.33 (br s, 2H), 4.20 (t, *J* = 6.0 Hz, 2H), 3.93 (br s, 2H), 3.55 (t, *J* = 7.1 Hz, 2H), 2.36 – 2.18 (m, 2H), 1.76 – 1.71 (m, 6H). ^13 ^C NMR (150 MHz, CDCl_3_) *δ* 195.24, 164.72, 161.27, 141.05, 140.18, 129.08, 117.75, 114.38, 112.85, 99.08, 66.84, 52.95, 51.38, 33.37, 28.56, 26.03, 25.44, 24.31. HRMS: calcd for C_18_H_23_N_2_O_2_S_2_ [M + H]^+^ 363.1195, found 363.1182.

#### 2.3.3. 4-((2-oxo-1,2-dihydroquinolin-7-yl)oxy)butyl piperidine-1-carbodithioate (4c)

Yield 84%; mp 151–152 °C; ^1^H NMR (600 MHz, CDCl_3_) *δ* 12.24 (s, 1H), 7.76 (d, *J* = 9.4 Hz, 1H), 7.46 (d, *J* = 8.6 Hz, 1H), 6.86 (d, *J* = 2.3 Hz, 1H), 6.83 (dd, *J* = 8.6, 2.3 Hz, 1H), 6.58 (d, *J* = 9.4 Hz, 1H), 4.32 (br s, 2H), 4.12 (t, *J* = 5.8 Hz, 2H), 3.92 (br s, 2H), 3.42 (t, *J* = 6.9 Hz, 2H), 2.07 – 1.87 (m, 4H), 1.75 – 1.70 (m, 6H). ^13 ^C NMR (150 MHz, CDCl_3_) *δ* 195.66, 164.89, 161.36, 140.94, 140.31, 129.04, 117.85, 114.26, 112.74, 99.05, 67.84, 52.88, 51.38, 36.76, 28.37, 25.54, 24.35. HRMS: calcd for C_19_H_25_N_2_O_2_S_2_ [M + H]^+^ 377.1352, found 377.1341.

#### 2.3.4. 5-((2-oxo-1,2-dihydroquinolin-7-yl)oxy)pentyl piperidine-1-carbodithioate (4d)

Yield 80%; mp 148–149 °C; ^1^H NMR (600 MHz, CDCl_3_) *δ* 11.61 (s, 1H), 7.78 (d, *J* = 9.4 Hz, 1H), 7.48 (d, *J* = 8.7 Hz, 1H), 6.85 (dd, *J* = 8.6, 2.3 Hz, 1H), 6.82 (d, *J* = 2.3 Hz, 1H), 6.58 (d, *J* = 9.4 Hz, 1H), 4.33 (br s, 2H), 4.09 (t, *J* = 6.4 Hz, 2H), 3.92 (br s, 2H), 3.54 – 3.24 (m, 2H), 1.92 – 1.80 (m, 5H), 1.76 – 1.64 (m, 7H). ^13 ^C NMR (150 MHz, CDCl_3_) *δ* 195.86, 164.48, 161.52, 141.04, 140.14, 129.12, 117.72, 114.26, 112.81, 98.97, 68.20, 52.88, 51.30, 36.98, 28.69, 28.56, 25.44, 24.35. HRMS: calcd for C_20_H_27_N_2_O_2_S_2_ [M + H]^+^ 391.1508, found 391.1527.

#### 2.3.5. 6-((2-oxo-1,2-dihydroquinolin-7-yl)oxy)hexyl piperidine-1-carbodithioate (4e)

Yield 85%; mp 118–120 °C; ^1^H NMR (600 MHz, DMSO-*d*_6_) *δ* 11.57 (s, 1H), 7.80 (d, *J* = 9.4 Hz, 1H), 7.55 (d, *J* = 9.4 Hz, 1H), 6.78 (d, *J* = 7.1 Hz, 2H), 6.29 (d, *J* = 9.4 Hz, 1H), 4.22 (br s, 2H), 4.00 (t, *J* = 6.5 Hz, 2H), 3.88 (br s, 2H), 3.24 (t, *J* = 7.2 Hz, 2H), 1.74 (q, *J* = 6.7 Hz, 2H), 1.71 – 1.60 (m, 4H), 1.56 (tq, *J* = 8.2, 5.3, 4.3 Hz, 4H), 1.48 – 1.38 (m, 4H). ^13 ^C NMR (150 MHz, DMSO-*d*_6_) *δ* 194.42, 162.70, 160.91, 141.13, 140.48, 129.71, 118.94, 113.72, 111.31, 98.99, 68.07, 52.67, 51.22, 36.60, 28.85, 28.52, 25.52, 24.06. HRMS: calcd for C_21_H_29_N_2_O_2_S_2_ [M + H]^+^ 405.1665, found 405.1685.

#### 2.3.6. 4-((2-oxo-1,2-dihydroquinolin-7-yl)oxy)butyl 4-methylpiperidine-1-carbodithio-ate (4f)

Yield 84%; mp 143–144 °C; ^1^H NMR (600 MHz, CDCl_3_) *δ* 12.54 (s, 1H), 7.76 (d, *J* = 9.4 Hz, 1H), 7.45 (d, *J* = 8.6 Hz, 1H), 6.86 (d, *J* = 2.2 Hz, 1H), 6.82 (dd, *J* = 8.7, 2.3 Hz, 1H), 6.57 (d, *J* = 9.4 Hz, 1H), 5.56 (br s, 1H), 4.63 (br s, 1H), 4.11 (t, *J* = 5.8 Hz, 2H), 3.42 – 3.40 (m, 2H), 3.18 (br s, 1H), 3.09 (br s, 1H), 1.98 – 1.92 (m, 4H), 1.79 – 1.74 (m, 3H), 1.29 (br s, 2H), 0.99 (d, *J* = 6.0 Hz, 3H). ^13 ^C NMR (150 MHz, CDCl_3_) *δ* 195.73, 165.06, 161.32, 140.87, 140.36, 129.00, 117.89, 114.22, 112.66, 99.03, 67.80, 52.11, 50.40, 36.81, 34.01, 33.52, 30.99, 28.38, 25.52, 21.30. HRMS: calcd for C_20_H_27_N_2_O_2_S_2_ [M + H]^+^ 391.1508, found 391.1534.

#### 2.3.7. 4-((2-oxo-1,2-dihydroquinolin-7-yl)oxy)butyl 4-isopropylpiperidine-1-carbodithi-oate (4 g)

Yield 80%; mp 126–127 °C; ^1^H NMR (600 MHz, CDCl_3_) *δ* 12.29 (s, 1H), 7.74 (d, *J* = 9.4 Hz, 1H), 7.45 (d, *J* = 8.6 Hz, 1H), 6.84 (d, *J* = 2.3 Hz, 1H), 6.82 (dd, *J* = 8.6, 2.4 Hz, 1H), 6.57 (d, *J* = 9.4 Hz, 1H), 4.38 (s, 2H), 4.11 (t, *J* = 5.8 Hz, 2H), 3.98 (s, 2H), 3.43 (t, *J* = 6.8 Hz, 2H), 2.77 (p, *J* = 6.4 Hz, 1H), 2.62 (br s, 4H), 2.05 – 1.87 (m, 4H), 1.84 (s, 1H), 1.08 (s, 3H), 1.07 (s, 3H). ^13 ^C NMR (150 MHz, CDCl_3_) *δ* 196.57, 164.95, 161.29, 140.83, 140.35, 129.02, 117.97, 114.19, 112.59, 99.01, 67.77, 54.39, 51.44, 50.11, 48.16, 36.66, 28.36, 25.50, 18.42. HRMS: calcd for C_22_H_31_N_2_O_2_S_2_ [M + H]^+^ 419.1821, found 420.1809.

#### 2.3.8. 4-((2-oxo-1,2-dihydroquinolin-7-yl)oxy)butyl [1,4′-bipiperidine]-1′-carbodithio -ate (4 h)

Yield 79%; mp 129–130 °C; ^1^H NMR (600 MHz, CDCl_3_) *δ* 12.39 (s, 1H), 7.75 (d, *J* = 9.4 Hz, 1H), 7.46 (d, *J* = 8.6 Hz, 1H), 6.85 – 6.81 (dd, *J* = 8.1, 2.5 Hz, 2H), 6.57 (d, *J* = 9.5 Hz, 1H), 5.60 (s, 1H), 4.69 (s, 1H), 4.11 (t, *J* = 5.9 Hz, 2H), 3.41 (s, 2H), 3.14 (br s, 2H), 2.64 (s, 1H), 2.52 (s, 4H), 2.02 – 1.83 (m, 6H), 1.60 (br s, 6H), 1.45 (br s, 2H). ^13 ^C NMR (150 MHz, CDCl_3_) *δ* 196.08, 165.00, 161.29, 140.82, 140.37, 129.02, 118.00, 114.19, 112.57, 99.01, 67.78, 62.07, 51.12, 50.26, 49.29, 36.94, 28.36, 27.97, 27.33, 26.30, 25.50, 24.65. HRMS: calcd for C_24_H_34_N_3_O_2_S_2_ [M + H]^+^ 460.2087, found 460.2129.

#### 2.3.9. 4-((2-oxo-1,2-dihydroquinolin-7-yl)oxy)butyl 4-hydroxypiperidine-1-carbodithio -ate (4i)

Yield 89%; mp 197–198 °C; ^1^H NMR (600 MHz, DMSO-*d*_6_) *δ* 11.58 (s, 1H), 7.81 (d, *J* = 9.4 Hz, 1H), 7.55 (d, *J* = 9.0 Hz, 1H), 6.80 – 6.77 (m, 2H), 6.29 (d, *J* = 9.4 Hz, 1H), 4.89 (d, *J* = 4.2 Hz, 1H), 4.56 (s, 1H),4.11 (br s, 1H), 4.03 (t, *J* = 6.0 Hz, 2H), 3.94 (br s, 1H), 3.84 – 3.81(m, 1H), 3.69 (br s, 1H), 3.31 (t, *J* = 7.1 Hz, 2H), 1.87 – 1.77 (m, 6H), 1.45 – 1.39 (m, 2H). ^13 ^C NMR (150 MHz, DMSO-*d*_6_) *δ* 194.56, 162.68, 160.80, 141.09, 140.47, 129.71, 118.98, 113.76, 111.26, 99.06, 67.69, 64.94, 49.03, 47.46, 36.50, 34.46, 33.94, 28.25, 25.68. HRMS: calcd for C_19_H_25_N_2_O_3_S_2_ [M + H]^+^ 393.1301, found 393.1328.

#### 2.3.10. 4-((2-oxo-1,2-dihydroquinolin-7-yl)oxy)butyl 4-(hydroxymethyl)piperidine-1-ca -rbodithioate (4j)

Yield 87%; mp 214–215 °C; ^1^H NMR (600 MHz, DMSO-*d*_6_) *δ* 11.58 (s, 1H), 7.81 (d, *J* = 9.5 Hz, 1H), 7.56 (d, *J* = 9.5 Hz, 1H), 6.81 – 6.77 (m, 2H), 6.30 (d, *J* = 9.4 Hz, 1H), 5.32 – 5.30 (m, 1H), 4.56 – 4.48 (m, 2H), 4.03 (t, *J* = 6.1 Hz, 2H), 3.32 – 3.26 (m, 4H), 3.17 (t, *J* = 13.4 Hz, 1H), 1.90 – 1.73 (m, 7H), 1.19 – 1.13 (m, 2H). ^13 ^C NMR (150 MHz, DMSO-*d*_6_) *δ* 194.34, 162.68, 160.81, 141.09, 140.47, 129.71, 118.98, 113.76, 111.26, 99.06, 67.70, 65.40, 51.72, 50.20, 38.36, 36.36, 29.17, 28.56, 28.27, 25.70. HRMS: calcd for C_20_H_27_N_2_O_3_S_2_ [M + H]^+^ 407.1457, found 407.1494.

#### 2.3.11. 4-((2-oxo-1,2-dihydroquinolin-7-yl)oxy)butyl 4-methylpiperazine-1-car-bodithioate (4k)

Yield 86%; mp 182–184 °C; ^1^H NMR (600 MHz, CDCl_3_) *δ* 12.29 (s, 1H), 7.74 (d, *J* = 9.4 Hz, 1H), 7.45 (d, *J* = 8.6 Hz, 1H), 6.84 (d, *J* = 2.3 Hz, 1H), 6.82 (dd, *J* = 8.6, 2.4 Hz, 1H), 6.57 (d, *J* = 9.4 Hz, 1H), 4.39 (br s, 2H), 4.11 (t, *J* = 5.7 Hz, 2H), 3.99 (br s, 2H), 3.42 (t, *J* = 6.8 Hz, 2H), 2.52 – 2.46 (m, 4H), 2.35 (br s, 3H), 2.00 – 1.90 (m, 4H). ^13 ^C NMR (150 MHz, CDCl_3_) δ 197.05, 164.95, 161.28, 140.83, 140.35, 129.03, 117.97, 114.20, 112.58, 99.01, 67.76, 54.41, 50.30, 49.61, 45.64, 36.73, 28.36, 25.48. HRMS: calcd for C_19_H_26_N_3_O_2_S_2_ [M + H]^+^ 392.1461, found 392.1498.

#### 2.3.12. 4-((2-oxo-1,2-dihydroquinolin-7-yl)oxy)butyl morpholine-4-carbodithioate (4 l)

Yield 88%; mp 156–158 °C; ^1^H NMR (600 MHz, CDCl_3_) *δ* 12.46 (s, 1H), 7.77 (d, *J* = 9.4 Hz, 1H), 7.46 (d, *J* = 8.6 Hz, 1H), 6.87 (d, *J* = 2.4 Hz, 1H), 6.82 (dd, *J* = 8.6, 2.4 Hz, 1H), 6.58 (d, *J* = 9.3 Hz, 1H), 4.37 (br s, 2H), 4.12 (t, *J* = 5.6 Hz, 2H), 3.98 (br s, 2H), 3.78 (br s, 4H), 3.44 (t, *J* = 6.7 Hz, 2H), 2.00 – 1.94 (m, 4H). ^13 ^C NMR (150 MHz, CDCl_3_) *δ* 197.65, 165.17, 161.30, 140.97, 140.33, 129.02, 114.29, 112.72, 99.07, 67.75, 66.41, 51.28, 50.43, 36.59, 28.35, 25.47. HRMS: calcd for C_18_H_23_N_2_O_3_S_2_ [M + H]^+^ 379.1144, found 379.1186.

#### 2.3.13. 4-((2-oxo-1,2-dihydroquinolin-7-yl)oxy)butyl pyrrolidine-1-carbodithioate (4 m)

Yield 86%; mp 170–172 °C; ^1^H NMR (600 MHz, CDCl_3_) *δ* 12.26 (s, 1H), 7.75 (d, *J* = 9.3 Hz, 1H), 7.46 (d, *J* = 8.6 Hz, 1H), 6.85 (d, *J* = 2.3 Hz, 1H), 6.83 (dd, *J* = 8.6, 2.3 Hz, 1H), 6.59 (d, *J* = 9.4 Hz, 1H), 4.11 (t, *J* = 5.8 Hz, 2H), 3.96 (t, *J* = 7.2 Hz, 2H), 3.67 (t, *J* = 6.6 Hz, 2H), 3.42 (t, *J* = 6.9 Hz, 2H), 2.12 – 2.05 (m, 2H), 2.02 – 1.87 (m, 6H). ^13 ^C NMR (150 MHz, CDCl_3_) *δ* 192.80, 164.86, 161.34, 140.91, 140.30, 129.03, 117.84, 114.24, 112.69, 99.03, 67.82, 54.97, 50.63, 36.02, 28.28, 26.04, 25.69, 24.31. HRMS: calcd for C_18_H_23_N_2_O_2_S_2_ [M + H]^+^ 363.1195, found 363.1234.

#### 2.3.14. 4-((4-methyl-2-oxo-1,2-dihydroquinolin-7-yl)oxy)butylpiperidine-1-carbodithioate (9a)

Yield 84%; mp 168–169 °C; ^1^H NMR (600 MHz, CDCl_3_) *δ* 7.60 (d, *J* = 8.8 Hz, 1H), 6.86 (d, *J* = 8.8 Hz, 1H), 6.80 (s, 1H), 6.46 (s, 1H), 4.33 (br s, 2H), 4.13 (t, *J* = 5.9 Hz, 2H), 3.92 (br s, 2H), 3.43 (t, *J* = 6.9 Hz, 2H), 2.50 (s, 3H), 2.00 – 1.94 (m, 4H), 1.75 – 1.71 (m, 6H). ^13 ^C NMR (150 MHz, CDCl_3_) *δ* 195.63, 161.17, 149.59, 139.71, 125.89, 114.87, 112.44, 99.29, 67.85, 52.93, 51.33, 36.73, 28.35, 25.55, 24.35, 19.24. HRMS: calcd for C_20_H_27_N_2_O_2_S_2_ [M + H]^+^ 391.1508, found 391.1529.

#### 2.3.15. 4-((3,4-dimethyl-2-oxo-1,2-dihydroquinolin-7-yl)oxy)butylpiperidine-1-carbodithioate (9b)

Yield 85%; mp 136–138 °C; ^1^H NMR (600 MHz, CDCl_3_) *δ* 7.64 (d, *J* = 9.0 Hz, 1H), 6.86 (dd, *J* = 9.0, 2.4 Hz, 1H), 6.70 (d, *J* = 2.5 Hz, 1H), 4.33 (br s, 2H), 4.11 (t, *J* = 6.0 Hz, 2H), 3.92 (br s, 2H), 3.43 (t, *J* = 7.1 Hz, 2H), 2.47 (s, 3H), 2.28 (s, 3H), 2.08 – 1.91 (m, 4H), 1.77 – 1.71 (m, 6H). ^13 ^C NMR (150 MHz, CDCl_3_) *δ* 195.61, 163.42, 160.13, 144.22, 137.59, 125.76, 115.33, 112.11, 98.89, 67.76, 52.89, 51.26, 36.72, 29.72, 28.36, 25.56, 24.34, 15.49, 12.55. HRMS: calcd for C_21_H_29_N_2_O_2_S_2_ [M + H]^+^ 405.1665, found 405.1724.

### 2.4. Biological evaluation

#### 2.4.1. *In vitro* inhibition experiments of ChEs

The *in vitro* inhibitory activities of test compounds against AChE and BuChE were determined by the spectrophotometric method of Ellman^25^. Acetylcholinesterase (AChE, from electric eel and human erythrocytes), butyrylcholinesterase (BuChE, from equine serum), S-butyrylthiocholine iodide (BTCI), acetylthiocholine iodide (ATCI), 5, 5′-dithiobis-(2-nitrobenzoic acid) (Ellman's reagent, DTNB) and the reference compounds (tarcine, donepezil and galanthamine) were obtained from Sigma-Aldrich (St. Louis, MO, USA). The compounds were first prepared in DMSO and then diluted with Tris-HCl buffer (50 mM, pH = 8.0, 0.1 M NaCl, 0.02 M MgCl_2_·6H_2_O) to yield corresponding test concentration (DMSO <0.01%). For each assay, 160 μL of 1.5 mM DTNB, 50 μL of AChE (0.22 U/mL for eeAChE; 0.05 U/mL for *h*AChE) or *eq*BuChE (0.12 U/mL) were incubated with 10 μL of different concentrations of test compounds at 37 °C for 6 min. After this, 30 μL of substrate (15 mM, ATCI or BTCI) was added, and the absorbance was measured with a wavelength of 405 nm at different time intervals (0, 60, 120, and 180 s). The concentration of each compound was tested in triplicate, and IC_50_ values were calculated graphically from percent inhibition curves, using the Graph Pad Prism 4.03 software (San Diego, CA, USA).

#### 2.4.2. *In vitro* blood-brain barrier permeation assay

The parallel artificial membrane permeation assay (PAMPA) for blood-brain-barrier was performed to predict the BBB penetration of test compounds^26^. Before the experiments, all compounds were prepared in DMSO, and the stock solutions were diluted in PBS/EtOH (70:30) to make secondary stock solutions (25 μg/mL). After the pre-treatment, the filter membrane on the 96-well filtration plate (PVDF membrane, pore size 0.45 mm, Millipore) was coated with 4 μL of PBL (Avanti Polar Lipids) in dodecane (20 mg/mL, Sigma-Aldrich). Then, 300 μL of PBS/EtOH (70:30) and 200 μL of diluted solution containing the corresponding drugs or test compounds were added to corresponding acceptor well and donor well, respectively. Afterwards, the acceptor filter plate was carefully placed on the donor plate to make the coated membrane touch both donor solution and acceptor buffer. After incubation for 18 h at 25 °C, the concentrations of test compounds in reference, acceptor and donor wells were determined by a UV plate reader (SpectraMax Plus 384, Molecular Devices). *P_e_* was calculated by the following expression: *P_e_* = {−V_d_V_a_/[(V_d_ + V_a_)At]}ln (1-drugacceptor/drugequilibrium), where V_d_ is the volume of donor well, V_a_ is volume in acceptor well, A is the filter area, t is the permeation time, drug_acceptor_ is the absorbance obtained in the acceptor well and drug_equilibrium_ is the theoretical equilibrium absorbance. Each compound was analysed at five wavelengths in four wells at least three independent runs, and the results were expressed as mean ± SD.

#### 2.4.3. Kinetic study of AChE inhibition

To determine the inhibition mechanism of present compounds, compound **4c** was taken for kinetic analysis by Ellman’s method^25^. Three concentrations of **4c** were selected for the study, and *h*AChE was chosen as enzyme source. Following a similar method mentioned above in enzyme inhibition assay, Lineweaver–Burk reciprocal plots were constructed by plotting 1/velocity against 1/[substrate] at varying concentrations of the substrate acetylthiocholine (0.05 – 0.5 mM). All measurements were performed in triplicate and data analysis was performed with Graph Pad Prism 4.03 software (San Diego, CA, USA).

#### 2.4.4. Molecular docking studies

The molecular docking studies were performed using the Molecular Operating Environment (Montreal, Canada, version 2015.10) software package. The crystal structures of *h*AChE (PDB code: 4EY7), *Tc*AChE (PDB code: 2CKM) and Aβ_1-42_ (PDB code: 1IYT) were downloaded from PBD. All water molecules from the protein were removed, and hydrogen atoms were added. The ligands were constructed using the builder module in MOE and were further minimised via MMFF94X force field. After that, the optimised geometry of ligand was saved as a (.mdb) file and docked into the binding pocket of the protein using the MOE-Dock programme. The London dG and Rigid Receptor were selected as initial and final scoring methods, respectively. The best 5 poses of each ligand were retained and scored. The MOE's pose viewer utility was used to analyse the geometry of resulting complex.

#### 2.4.5. Inhibition of AChE-induced Aβ_1-42_ aggregation

The inhibitory activities of compounds **4c-f** and **9a-b** on AChE-induced Aβ_1-42_ aggregation were evaluated by a Thioflavin T (ThT)-binding assay^27^^,^^28^. 1, 1, 1, 3, 3, 3-hexafluoro-2-propanol (HFIP) and Thioflavin T (ThT) were obtained from TCI (Shanghai) Development. β-Amyloid_1-42_ (Aβ_1-42_), supplied as trifluoroacetate salt, and eeAChE were purchased from Royobiotech Co., Ltd (Shanghai, China) and Sigma-Aldrich (St. Louis, MO, USA), respectively. For co-incubation experiments, aliquots of Aβ_1-42_ peptide and AChE in presence or absence of the test compounds were incubated for 48 h at 37 °C. The final concentrations of Aβ (dissolved in DMSO and diluted 0.215 M sodium phosphate buffer, pH 8.0), AChE (dissolved in 0.215 M sodium phosphate buffer, pH 8.0) and test compounds were 200 μM, 2 μM and 100 μM, respectively. After co-incubation, 200 μL of 5 μM ThT in 50 mM glycine-NaOH buffer (pH 8.0) was added, and the fluorescence was determined on a multi-mode plate reader (SpectraMax M5, Molecular Devices, Sunnyvale, CA, USA) at λ_ex_ = 446 nm and λ_em_ = 490 nm, respectively. The percent inhibition of the AChE-induced Aβ aggregation due to the presence of inhibitor was calculated by the following formula: 100 – (IF_i_/IF_o_*100), where IF_i_ and IF_o_ are the fluorescence intensities obtained for Aβ_1-42_ aggregation in the presence and in the absence of inhibitor, respectively. Each assay was run in triplicate and each reaction was repeated at least three independent times.

#### 2.4.6. Inhibition of self-induced Aβ_1-42_ aggregation

The inhibitory activities of compounds **4c-f** and **9a-b** on self-induced Aβ_1-42_ aggregation were also measured by the ThT assay^29^. HFIP and ThT were purchased from TCI (Shanghai) Development. Aβ_1-42_ was obtained from Royobiotech Co., Ltd (Shanghai, China). The monomeric Aβ_1-42_ samples were prepared by HFIP and diluted with a 50 mM phosphate buffer (pH 7.4) to give a 25 μM solution. The test compound was dissolved in DMSO (250 μM) for storage and needed not to be diluted prior to use. In each well, 1 μL of test compound at final concentration of 25 μM and 9 μL of Aβ_1-42_ sample were added, and the obtained mixture was incubated in dark at room temperature for 46–48 h with no agitation. Then, 200 μL of 5 μM ThT in 50 mM glycine-NaOH buffer (pH 8.0) was added, and the percent inhibition was calculated by the method same as that of AChE-induced Aβ_1–42_ experiment.

#### 2.4.7. Cytotoxicity study of human neuroblastoma SH-SY5Y cells

The cytotoxicity of test compound on the human neuroblastoma SH-SY5Y cells was evaluated by MTT assay^30^. The SH-SY5Y cells were grown in a 1:1 mixture of Eagle's minimum essential medium (EMEM) and ham's F-12 medium supplemented with 10% foetal bovine serum (FBS), 100 U/mL penicillin and 100 mg/mL streptomycin in 5% CO_2_ at 37 °C. For assay, cells were seeded into 96-well plates at a density of 10000 cells/well and treated with compound **4c** for 24 h. After this treatment, 20 μL of MTT at 37 °C was added. Four hours later, the medium was removed, and 200 μL of DMSO was added to dissolve the formazan crystal formed. The absorbance was measured using a microculture plate reader with a test wavelength of 570 nm and a reference wavelength of 630 nm. Results are expressed as the mean ± SD of three independent experiments.

#### 2.4.8. Acute oral toxicity studies

A total of 40 Kunming mice (half male and half female) were used to determine the acute toxicity of compound **4c**^31^^,^^32^. All mice weighting 18–25 g were obtained from Hunan SJA Laboratory Animal Co., Ltd. and randomly divided into corresponding groups. They were maintained under a 12 h light/dark cycle and allowed free access to tap water and standard laboratory chow. The room was maintained at temperature of 23 ± 2 °C with a relative humidity of 55 ± 5%. Prior to dosing, all animals were acclimated to this environment for 5 days. Compound **4c** was suspended in 0.5% carboxymethyl cellulose sodium (CMC-Na) salt solution and given via oral administration to the divided experimental groups (0, 625, 1250 and 2500 mg/kg, *n* = 10 per group). After administration, animals were observed continuously for the first 4 h for any abnormal behavioural changes or deaths, then intermittently for the next 24 h, and occasionally thereafter for 14 days for the onset of any delayed effects. On the 14th day, all mice were sacrificed after being anaesthetized by ether, and the possible toxic damage to liver, heart and kidneys was examined macroscopically.

## 3. Results and discussion

### 3.1. Chemistry

**Scheme 1:**
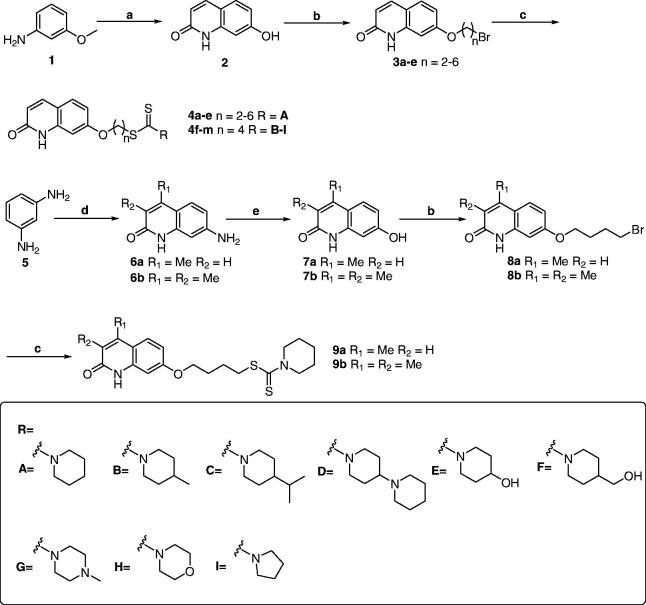
Synthesis of compounds **4a-m** and **9a-b**. Reagents and conditions: (**a**) (i) cinnamoyl chloride, dry dichloromethane, 4 h, reflux; (ii) AlCl_3_, chlorobenzene, 8 h, reflux; (**b**) α, ω-dibromoalkanes, K_2_CO_3_, acetone, reflux, 4 h; (**c**) appropriate secondary amines, CS_2_, TEA, DMF, r.t., 12 h. (**d**) ethyl acetoacetate for **6a**, ethyl 2-methylacetoacetate for **6 b**, 150 °C, 48 h; (**e**) (i) NaNO_2_, H_2_SO_4_, 0 °C; (ii) 10 M, H_2_SO_4_, reflux, 10 min.

The synthetic pathways of target derivatives are illustrated in Scheme 1. According to the previously reported methods, the 2-quinolinone cores were obtained through different procedures and starting materials^20^^,^^23^^,^^24^. The compound **2** was prepared from the condensation of *trans*-cinnamic acid chloride with *m*-anisidine, followed by an intramolecular Friedel-Crafts acylation/dearylation reaction in refluxing chlorobenzene with aluminium chloride as Lewis acid, while the compounds 7**a-b** were prepared from *m*-phenylenediamine and ethyl acetoacetate/ethyl 2-methylacetoacetate by three steps including Pechmann condensation reaction, diazotisation, and hydrolysis. When these key intermediates were finished, they were treated with corresponding ɑ, ω-dibromoalkanes to provide compounds **3a-e** and **8a-b**. Finally, reacting compounds **3a-e** and **8a-b** with the appropriate secondary amines, carbon disulphide and TEA in DMF afforded the target compounds **4a-m** and **9a-b**.

### 3.2. Biological evaluation

#### 3.2.1. *In vitro* inhibition studies of AChE and BuChE

The inhibitory activities of target compounds **4a-m** and **9a-b** against *ee*AChE (from electric eel) and *eq*BuChE (from equine serum) were determined according to the spectrophotometric method described by Ellman et al.^25^. For comparison purposes, three well-known cholinesterase inhibitors, donepezil, galanthamine and tacrine, were used as reference compounds. The experimental results were presented as IC_50_ (μM), or for poorly active compounds, as the percentage of inhibition at 10 μM. All these results are summarised in Table 1. It can be seen from the table that most of compounds show moderate to good inhibitory activity on AChE with IC_50_ values ranging from micromolar to sub-micromolar. Among these compounds, compound **4c** displayed the highest inhibitory activity to AChE with IC_50_ value of 0.22 μM, which was similar to that of tacrine (IC_50_ = 0.21 μM) and 7.5 times more potent than that of galanthamine (IC_50_ = 1.65 μM). With exception of compound **4 h**, no significant BuChE inhibitory activity was observed for the present compounds. Considering inhibition of BuChE may lead to unexpected side effects in the peripheral tissues, these compounds will be more beneficial for AD treatment.

**Table 1.** Inhibition of *ee*AChE and *eq*BuChE by compounds **4a-m** and **9a-b**.

					*ee*AChE^a^	*eq*BuChE^b^
Compd.	n	R	R_1_	R_2_	IC_50_ (μM)/Inhibition (%)	IC_50_ (μM)/Inhibition (%)
4a	2	**A**	H	H	45.3 ± 0.35%	16.69 ± 2.36%
4b	3	**A**	H	H	3.96 ± 0.71 μM	16.60 ± 4.01%
4c	4	**A**	H	H	0.22 ± 0.03 μM	45.37 ± 8.34%
4d	5	**A**	H	H	0.24 ± 0.02 μM	20.95 ± 2.61%
4e	6	**A**	H	H	0.35 ± 0.02 μM	17.49 ± 2.17%
4f	4	**B**	H	H	0.69 ± 0.22 μM	37.22 ± 1.22%
4g	4	**C**	H	H	9.61 ± 1.12 μM	31.43 ± 3.15%
4h	4	**D**	H	H	3.80 ± 0.35 μM	2.00 ± 0.23 μM
4i	4	**E**	H	H	20.17 ± 3.27%	15.81 ± 2.45%
4j	4	**F**	H	H	14.52 ± 2.44%	23.91 ± 2.03%
4k	4	**G**	H	H	23.14 ± 5.54%	28.65 ± 3.44%
4l	4	**H**	H	H	12.31 ± 1.76%	30.47 ± 4.11%
4m	4	**I**	H	H	31.43 ± 1.65%	7.63 ± 0.92%
9a	4	**A**	Me	H	0.30 ± 0.12 μM	18.31 ± 3.15%
9b	4	**A**	Me	Me	0.34 ± 0.01 μM	21.20 ± 1.60%
Donepezil	–	–	–	–	0.041 ± 0.002 μM	4.22 ± 0.20 μM
Galanthamine	–	–	–	–	1.65 ± 0.14 μM	11.68 ± 3.49 μM
Tacrine	–	–	–	–	0.21 ± 0.02 μM	0.057 ± 0.013 μM

^a^The 50% inhibitory concentration of *ee*AChE or percent inhibition with inhibitor at 10 μM (means ± SD of three experiments).

^b^The 50% inhibitory concentration of *eq*BuChE or percent inhibition with inhibitor at 10 μM (means ± SD of three experiments).

The previous studies indicated that the linker length between PAS and CAS binding moieties played a crucial role in AChE inhibition^33–35^. Therefore, to determine the optimal length for the present compounds, compounds **4a-e** with different linker lengths (*n* = 2–6) were prepared in the initial step. As shown in the table, the inhibitory activities for AChE changed significantly as the length of the alkyl chain was varied. From the IC_50_ values, compound **4c** containing a four-carbon atom linker showed better inhibitory activity than the other compounds, which suggested that the suitable linker length between the two anchoring groups for AChE inhibition was four carbon atoms.

With the optimal length in hand, different terminal amine groups were introduced to investigate their possible influences on AChE inhibition. The no substituted piperidine moiety seemed to be essential for AChE inhibitory activity. Compound **4f** (IC_50_ = 0.69 μM) bearing a 4-methyl group on the piperidine ring showed 3-fold less inhibitory activity than the no substituted compound **4c** (IC_50_ = 0.22 μM). Replacement of the methyl group with an isopropyl group led to a largely drop in AChE inhibition. Compound **4 g** (IC_50_ = 9.61 μM) only gave a micromolar inhibition to AChE. However, it should be noted that introduction of another piperidine to 4-postion of piperidine ring resulted in a significant improvement in BuChE inhibition. Compound **4 h** (IC_50_ = 3.80 μM for AChE; IC_50_ = 2.00 μM for BuChE) was the only compound with BuChE inhibitory activity in this series. Installation of a hydroxy group on piperidine ring was not tolerated, as compounds **4i** (20.17% inhibition) and **4j** (14.52% inhibition) did not show any inhibitory activity on AChE. Besides, replacement of the piperidine group with other cyclic amines were also not beneficial for the AChE inhibition. Compounds **4k** (23.14% inhibition) and **4 l** (12.31% inhibition), possessing a 4-methylpiperazine moiety and a morpholinyl moiety, respectively, displayed neglected inhibition on AChE. Unlike our previous study that contraction of the piperidine ring to pyrrole ring had little impact on the inhibitory activity^21^, in present work compound **4 m** showed the inhibitory activity less than 50% at 10 μM. Moreover, compound **9a** (IC_50_ = 0.30 μM) with mono-methyl group and compound **9b** (IC_50_ = 0.34 μM) with dimethyl groups on coumarin ring were also synthesised to further extend the SARs. The results indicated that introduction of methyl group to coumarin slightly reduced the inhibitory activity on AChE in comparison to their analogue **4c**.

### 3.2.2. *In vitro* blood-brain barrier permeation assay

**Figure 2.**
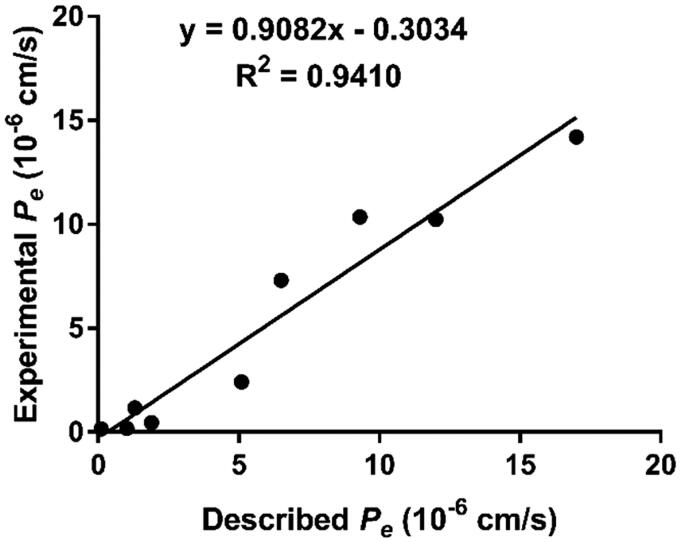
Lineal correlation between experimental and reported permeability of commercial drugs using the PAMPA-BBB assay. *P_e_* (exp.) = 0.9082*P_e_*(bibl.) – 0.3034 (R^2^ = 0.9410).

The ability to penetrate the blood-brain barrier (BBB) is the first requirement for successful central nervous system (CNS) drugs ^36^^,^^37^. Therefore, to determine the BBB penetration of our present compounds, a parallel artificial membrane permeability (PAMPA) assay was performed. This assay was established by Di et al. and has been successfully applied to predict the passive BBB permeation of a multitude of diverse compounds^26^. After comparing experimental permeabilities with the reported values of 9 commercial drugs, a plot of experiment data versus the bibliographic values gave a good linear correlation: *P_e_* (exp) = 0.9082 *P_e_* (bibl.) – 0.3034 (*R*^2 ^= 0.9410) (Figure 2 and Table 2). From this equation and considering the limit established by Di et al. for BBB permeation, we determined that compounds with permeabilities above 3.33 × 10^−6 ^cm s^−1^ could cross the BBB. As shown in the Table 3, with exception of compounds **4i** and **4j**, all compounds showed the *P_e_* value higher than 3.33, which suggested that they were able to cross the BBB and might reach the biological targets located in the CNS.

**Table 2.** Permeability *P_e_*(×10^−6^) in the PAMPA-BBB assay for 9 commercial drugs in the experiment validation.

Commercial drugs	Bibliography^a^	Experiment^b^
Testosterone	17.0	14.2 ± 3.6
Estradiol	12.0	10.2 ± 2.5
Progesterone	9.3	10.4 ± 1.9
Chlorpromazine	6.5	7.3 ± 2.3
Corticosterone	5.1	2.4 ± 0.8
Hydrocortisone	1.9	0.46 ± 0.12
Caffeine	1.3	1.2 ± 0.2
Atenolol	1.02	0.19 ± 0.23
Theophylline	0.1	0.16 ± 0.07

^a^Adapted from Di et al.^26^.

^b^Experimental data are expressed as mean ± SD from three independent experiments, using PBS: EtOH (70:30) as solvent.

**Table 3.** Permeability *P_e_* (×10^6^ cm/s) in the PAMPA-BBB assay for target compounds and their predicted penetration into CNS.

Compound	*P_e_* (×10^6^ cm/s)^a^	Prediction^b^
**4a**	8.57 ± 1.23	CNS+
**4b**	8.78 ± 1.11	CNS+
**4c**	8.91 ± 2.03	CNS+
**4d**	8.82 ± 1.34	CNS+
**4e**	9.85 ± 0.98	CNS+
**4f**	6.30 ± 0.86	CNS+
**4g**	7.14 ± 1.97	CNS+
**4h**	6.42 ± 1.02	CNS+
**4i**	2.83 ± 0.65	CNS±
**4j**	3.25 ± 0.82	CNS±
**4k**	4.54 ± 0.84	CNS+
**4l**	7.35 ± 1.05	CNS+
**4m**	10.00 ± 2.63	CNS+
**9a**	8.79 ± 2.10	CNS+
**9b**	8.67 ± 1.76	CNS+

^a^Permeability *P_e_* (×10^6^ cm/s) values are expressed as mean ± SD from three independent experiments, using PBS: EtOH (70:30) as solvent.

^b^CNS + is predicted as high BBB permeation with *P_e_* (×10^6^ cm/s) > 3.33, CNS ± is uncertain for BBB permeation with 1.51 < *P_e_* (×10^6^ cm/s) < 3.33.

### 3.2.3. *In vitro* inhibition studies of hAChE

Since almost all compounds could cross the BBB, we then selected compounds **4c-f** and **9a-b** with sub-micromolar inhibitory activity for further evaluation on *h*AChE. As shown in Table 4, all selected compounds having potent inhibition on eeAChE also exhibit high inhibition to *h*AChE. It is worth noting that compounds **4c**, **4e** and **9b** did not display better activity on *ee*AChE than tacrine but showed stronger inhibition on *h*AChE than tacrine, especially for compound **4c** (IC_50_ = 0.16 μM); it presented nearly 3-fold potent inhibitory activity on *h*AChE than tacrine (IC_50_ = 0.47 μM), which was largely improved in comparison to its inhibitory activity on eeAChE.

**Table 4.** Inhibition of *h*AChE, and AChE- and self-induced Aβ_1-42_ aggregation by selected compounds.

	*h*AChE	Inhibition of Aβ_1–42_ aggregation (%)	
Compd.	IC_50_ (μM)^a^	AChE-induced^b^	Self-induced^c^
4c	0.16 ± 0.02	29.02 ± 2.06	30.67 ± 6.54
4d	0.57 ± 0.01	24.11 ± 3.04	26.01 ± 3.01
4e	0.38 ± 0.02	20.28 ± 2.21	25.82 ± 1.43
4f	1.21 ± 0.33	12.06 ± 3.18	20.35 ± 3.22
9a	0.76 ± 0.10	14.19 ± 2.85	33.36 ± 4.85
9b	0.39 ± 0.01	24.37 ± 3.03	35.29 ± 2.17
Donepezil	0.021 ± 0.001	26.16 ± 2.55	–
Tacrine	0.47 ± 0.03	–	–
Curcumin	–	–	37.27 ± 2.76

^a^The 50% inhibitory concentration of hAChE (means ± SD of three experiments).

^b^Inhibition of AChE-induced Aβ_1–42_ aggregation. The thioflavin-T fluorescence method was used, and the concentration of the tested inhibitor was 100 μM (means ± SD of three experiments).

^c^Inhibition of self-induced Aβ_1-42_ aggregation. The thioflavin-T fluorescence method was used, and the measurements were carried out in the presence of 25 μM inhibitor (means ± SD of three experiments).

### 3.2.4. Kinetic study of hAChE inhibition

**Figure 3.**
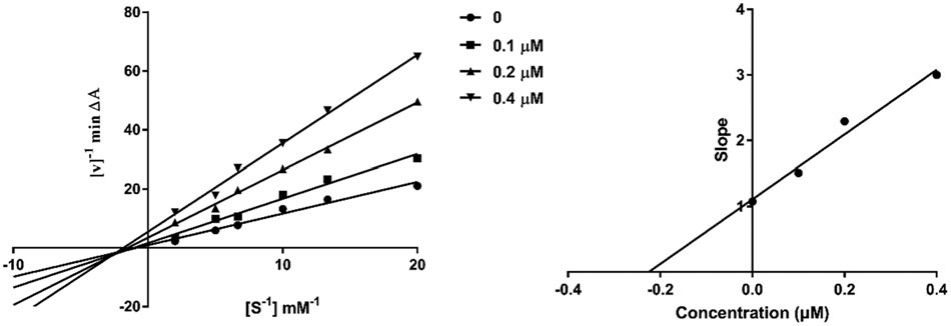
Kinetic study on the inhibition mechanism of *h*AChE by compound **4c**. (**a**) Overlaid Lineweaver-Burk reciprocal plots of *h*AChE initial velocity at increasing substrate concentrations (0.05–0.50 mM) in the absence of inhibitor and in the presence of different concentrations (0.4, 0.2 and 0.1 μM) of **4c** are shown. (**b**) The plot of the slopes of the Lineweaver-Burk plots versus inhibitor concentration.

To investigate the inhibition mechanism of present compounds with AChE, compound **4c**, the most potent inhibitor in this series, was taken as a representative for enzyme kinetic study. It can be seen from the Lineweaver-Burk plots (Figure 3(a)) that both slopes and intercepts are increased as the concentrations of the inhibitor are increased. This pattern indicated a mixed-type inhibition, which suggested that the present compounds might be dual binding site inhibitors, being able to bind simultaneously to the PAS and CAS of AChE. Replots of the slope versus concentration of **4c** gave an estimate of competitive inhibition constant, *Ki*, of 0.225 μM (Figure 3(b)).

### 3.2.5. Molecular modelling study with AChE

**Figure 4.**
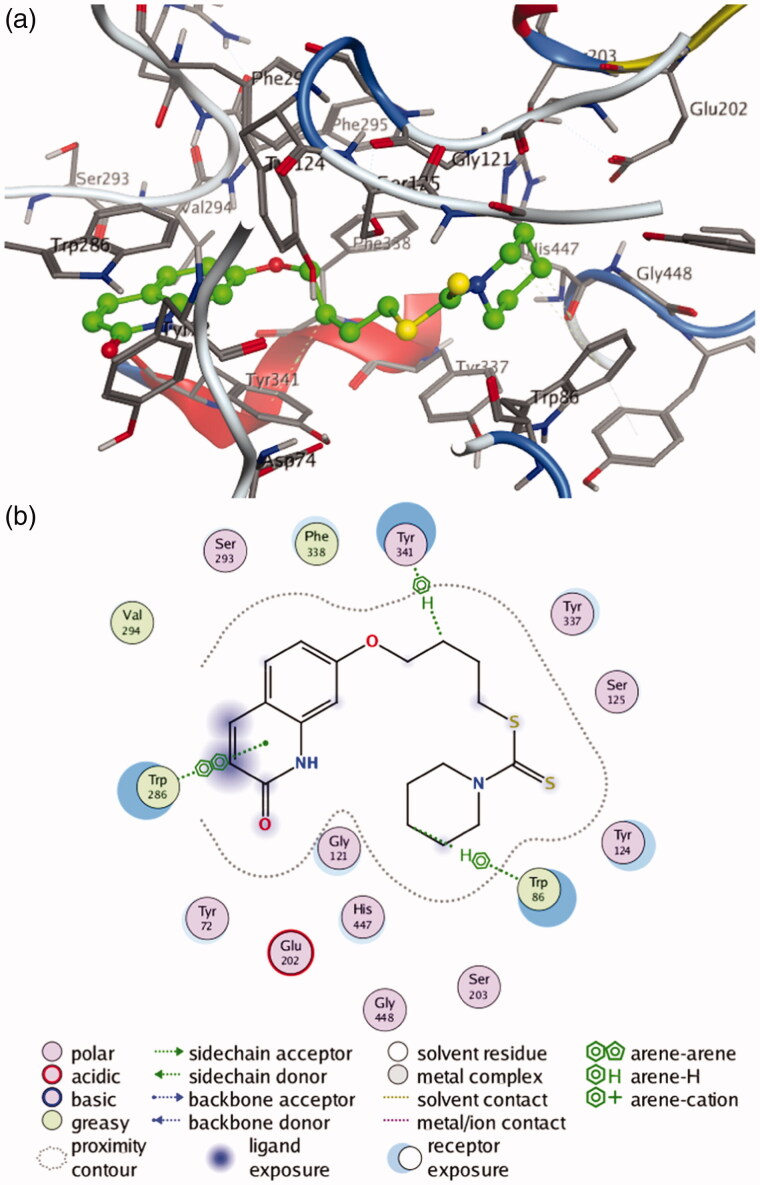
(**a**) 3 D docking model of compound **4c** with *h*AChE. Atom colours: green-carbon atoms of **4c**, gray-carbon atoms of residues of *h*AChE, dark blue-nitrogen atoms, red-oxygen atoms, yellow-sulfur atoms. (**b**) 2 D schematic diagram of docking model of compound **4c** with *h*AChE. The figure was prepared using the ligand interactions application in MOE.

To further determine the interaction mode of present compounds with AChE, molecular modelling studies were performed with a Molecular Operating Environment (MOE) software. The crystal structures of *h*AChE complexed with donepezil (PDB code 4EY7) and *Tc*AChE complexed with bis(7)-tacrine were used to establish the starting model. Donepezil was used as a reference compound. The docking results (see Supporting Information, Supplementary Table S1–S2) indicated that compounds **4c-f** and **9a-b** with potent inhibitory activities on both *h*AChE and *ee*AChE showed lower S value than the other compounds in corresponding crystal structure of AChE. Inspection of the docking orientations found that most of these compounds exhibited the binding modes with *h*AChE and *Tc*AChE similar to those of donepezil. The most potent compound **4c** showed the lowest S value on both *h*AChE and *Tc*AChE. As shown in Figure 4, the interaction mode of compound **4c** with *h*AChE indicate that compound **4c** fits well into the active site of *h*AChE, which can occupy the entire enzymatic CAS, mid-gorge site and PAS. The quinolinone moiety bound to the PAS, forming a π-π stacking interaction with Trp 286, while the terminal piperidine group located into the CAS and established a ‘arene-H’ interaction with Trp 86. In addition, the polymethylene chain was folded in a conformation to make it interact with the mid-gorge site through a ‘arene-H’ interaction with Tyr 341. All these results demonstrated that the present compounds were dual binding site inhibitors of AChE, which was in agreement with our results obtained from the kinetic analysis.

### 3.2.6. Inhibition of AChE-induced Aβ_1-42_ aggregation

It has been established that the PAS mediates AChE-triggered Aβ aggregation. Thus, compounds capable of interacting with the PAS may have the ability to reduce Aβ aggregation. Because our compounds have been demonstrated to be dual binding site inhibitors of AChE, compounds **4c-f** and **9a-b** with potent inhibitory activity on AChE were also selected for testing their inhibitory activities on AChE-induced Aβ aggregation by a thioflavin T (ThT) fluorescence method ^27^^,^^28^. It can be seen from the Table 4 that, in comparison to the reference compound donepezil, most compounds can efficiently inhibit the Aβ aggregation with inhibition rates ranging from 12.06% to 29.02% at 100 μM. Generally, compounds with potent inhibition on AChE also exhibited high inhibitory activity for Aβ aggregation. Compound **4c** that displayed the most potent inhibitory activity on both eeAChE and *h*AChE exerted the strongest effect on inhibition of Aβ aggregation. It showed the inhibition rate of 29.02%, which was more potent than that of donepezil (26.16% inhibition).

### 3.2.7. Inhibition of self-induced Aβ_1-42_ aggregation

The capacities of these selected compounds to inhibit self-induced Aβ_1–42_ aggregation were also investigated by employing the same ThT-based fluorometric assay^29^. Curcumin, a natural inhibitor of self-induced Aβ aggregation, was used as positive control. From the results summarised in Table 4, all compounds exhibited moderate inhibitory activity for Aβ aggregation at a concentration of 25 μM. The relatively long linkers were not beneficial for the inhibitory activity, as compounds **4d-f** showed lower activity than their short linker analogues **4c** and **9a-b**. Among them, compound **9b** with dimethyl groups on quinolinone ring presented the most potent inhibitory activity for Aβ aggregation with inhibition rate of 35.29% at 25 μM.

### 3.2.8. Molecular modelling study with Aβ_1-42_

**Figure 5.**
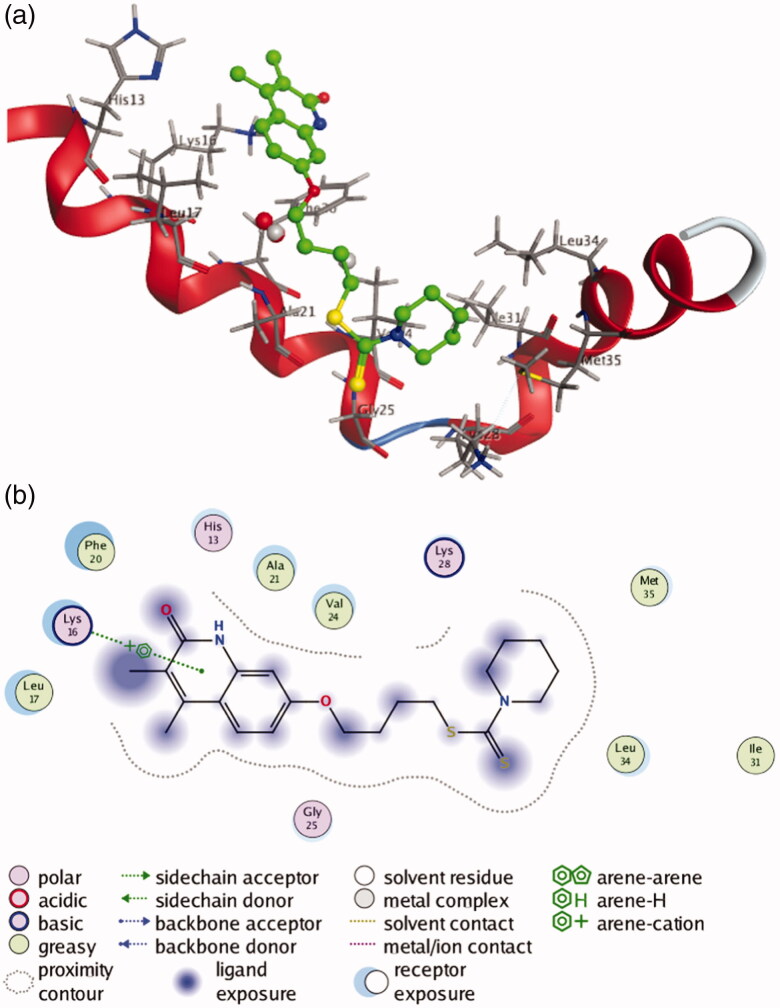
(**a**) 3 D docking model of compound **9b** with Aβ_1-42_. Atom colours: green-carbon atoms of **9b**, gray-carbon atoms of residues of Aβ, dark blue-nitrogen atoms, red-oxygen atoms, yellow-sulfur atoms. (**b**) 2 D schematic diagram of docking model of compound **9b** with Aβ. The figure was prepared using the ligand interactions application in MOE.

Since most of the selected compounds could efficiently inhibit Aβ_1-42_ aggregation, their possible binding modes with Aβ_1-42_ were also investigated by molecular docking studies. The studies were performed on the Molecular Operating Environment (MOE) software, and compound **9b** with most potent inhibitory activity for self-induced Aβ_1-42_ aggregation was chosen for docking into the structure of Aβ_1-42_ (PDB code 1IYT). As shown in Figure 5, compound **9b** is transversely fitted along the major helix, and forms a π-cation interaction between its quinolinone ring and Lys 16 of Aβ_1-42_. Hydrophobic interactions also play an important role in the stability of **9b**/Aβ_1-42_ complex. From the Figure 5(a), it can be seen that compound **9b** establishes hydrophobic interactions with residues Leu 17, Phe 20, Ala 21, Val 24, Ile 31, Leu 34 and Met 35.

### 3.2.9. Cytotoxicity on human neuroblastoma SH-SY5Y cells

**Figure 6.**
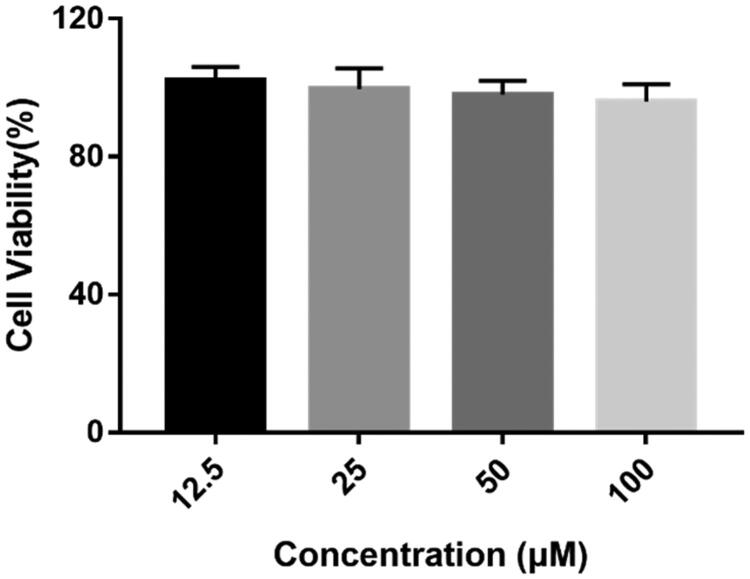
Cytotoxicity of compound **4c** on human neuroblastoma cells SH-SY5Y. SH-SY5Y cells were incubated with different concentrations of compound **4c** (12.5–100 µM) for 24 h. The results are shown as the percentage of viable cells after treatment with compound **4c** *vs* untreated control cells. Date are expressed as mean ± SD from three independent experiments.

After all above evaluation, compound **4c** with potent inhibitory activity for both *ee*AChE and *h*AChE as well as good BBB permeability and Aβ aggregation inhibitory effect was considered as the most promising compound for further study. To determine whether compound **4c** have potential toxicity on nerve cells, its cytotoxicity on human neuroblastoma SH-SY5Y cells was measured by the 3–(4,5-dimethylthiazol-2-yl)-2, 5-diphenyltetrazolium (MTT) assay^30^. As shown in Figure 6, compound **4c** did not show a significant effect on cell viability at doses up to 100 μM, which indicated that it might be a safe agent for treating AD.

### 3.2.10. Acute toxicity

Toxicity of new chemical entity is a major obstacle in path of drug discovery and development^31^^,^^32^^,^^38^. Therefore, compound **4c** was select for acute toxicity study in vivo to further investigate its safety property. After delivering compound **4c** to Kunming (KM) mice with different dosages (625, 1250 and 2500 mg/kg) by oral administration, no evident toxicity signs such as death, significant weight loss, and drastically altered consumption of water or food, were observed during the two-week experimental period. Besides, at the end of the experiment, all used animals were sacrificed, and the possible toxic damage on their organs was also examined. The results indicated that compound **4c** did not cause any toxicity effect at a dose up to 2500 mg/kg.

## 4. Conclusion

A series of novel quinolinone derivatives containing dithiocarbamate moiety were designed and synthesised as multifunctional AChE inhibitors for the treatment of AD. Most of these compounds exhibited potent *ee*AChE inhibitory activity and excellent selectivity to *ee*AChE over *eq*BuChE. Among them, compound **4c** having a four-carbon atom linker and a piperidine moiety at terminal position displayed the highest activity to inhibit *ee*AChE, and it was also the strongest inhibitor of *h*AChE with IC_50_ value of 0.16 μM, which was nearly 3-fold more potent than that of tacrine (IC_50_ = 0.47 μM). Kinetic and molecular docking analyses revealed that compound **4c** was a mix-typed inhibitor able to bind simultaneously to PAS and CAS of *h*AChE. Besides, compound **4c** presented the best ability to inhibit AChE-induced Aβ aggregation (29.02% at 100 μM) and could efficiently inhibit self-induced Aβ aggregation (30.67% at 25 μM). More importantly, it showed negligible toxicity to SH-SY5Y cells, good ability to penetrate the BBB and no significant acute toxicity in mice at doses up to 2500 mg/kg. Overall, we propose that compound **4c** is a promising candidate deserved for further study.

## Supplementary Material

Supplemental Material
