# Supplementary material for: Design, synthesis and evaluation of quinolinone derivatives containing dithiocarbamate moiety as multifunctional AChE inhibitors for the treatment of Alzheimer’s disease
Source: J Enzyme Inhib Med Chem. 2019 Nov 7;35(1):118–28. doi: 10.1080/14756366.2019.1687460 (PMC6844382; doi:10.1080/14756366.2019.1687460)
Supplement: Supplemental Material [file IENZ_A_1687460_SM8197.pdf]

Supporting Information

Design, synthesis and evaluation of quinolinone derivatives containing dithiocarbamate moiety as multifunctional AChE inhibitors for the treatment of Alzheimer's disease

Jie Fu[a,b], Fengqi Bao[b], Min Gu[b], Jing Liu[d], Zhipeng Zhang[c], Jiaoli Ding[c], Sai-Sai Xie[c]*, Jinsong Ding[a]*

[a]Xiangya School of Pharmaceutical Sciences, Central South University, Changsha, Hunan, China.

[b]Jiangsu sinomune Pharmaceutical Co., Ltd., 35 Jingxin Road, Xibei Town Industrial Park, Xishan District, Wuxi, Jiangsu, China.

[c]National Pharmaceutical Engineering Center for Solid Preparation in Chinese Herbal Medicine, Jiangxi University of Traditional Chinese Medicine, Nanchang, China

[d]School of Pharmacy, Jiangxi University of Traditional Chinese Medicine, Nanchang, PR China

*Authors for correspondence: xiesaisainanchang@hotmail.com (Sai-Sai Xie)

*Authors for correspondence: dingjs0221@163.com (Jinsong Ding)

Contents

1. ***The ^{1}H NMR, ^{13}C NMR and HRMS of the target compounds 4a-m and 9a-b. S2***

2. Docking results of compounds 4a-m and 9a-b with hAChE and TcAChE. S32

[1] H NMR spectrum of compound **4a**

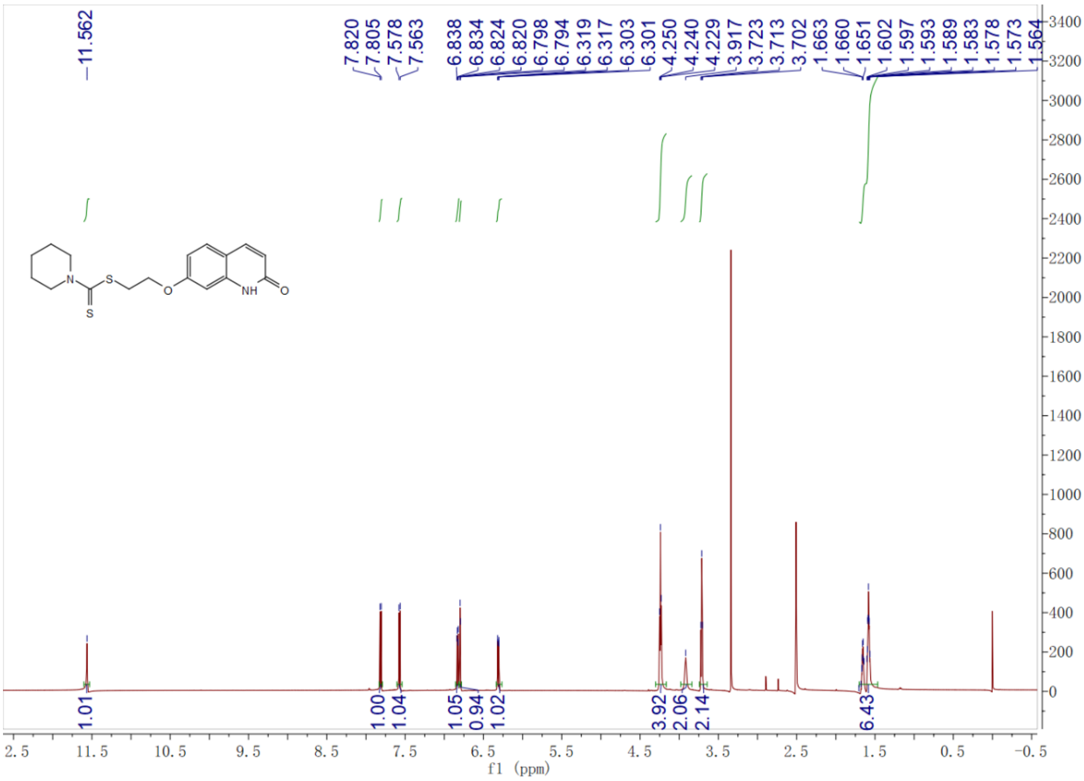

[13] C NMR spectrum of compound **4a**

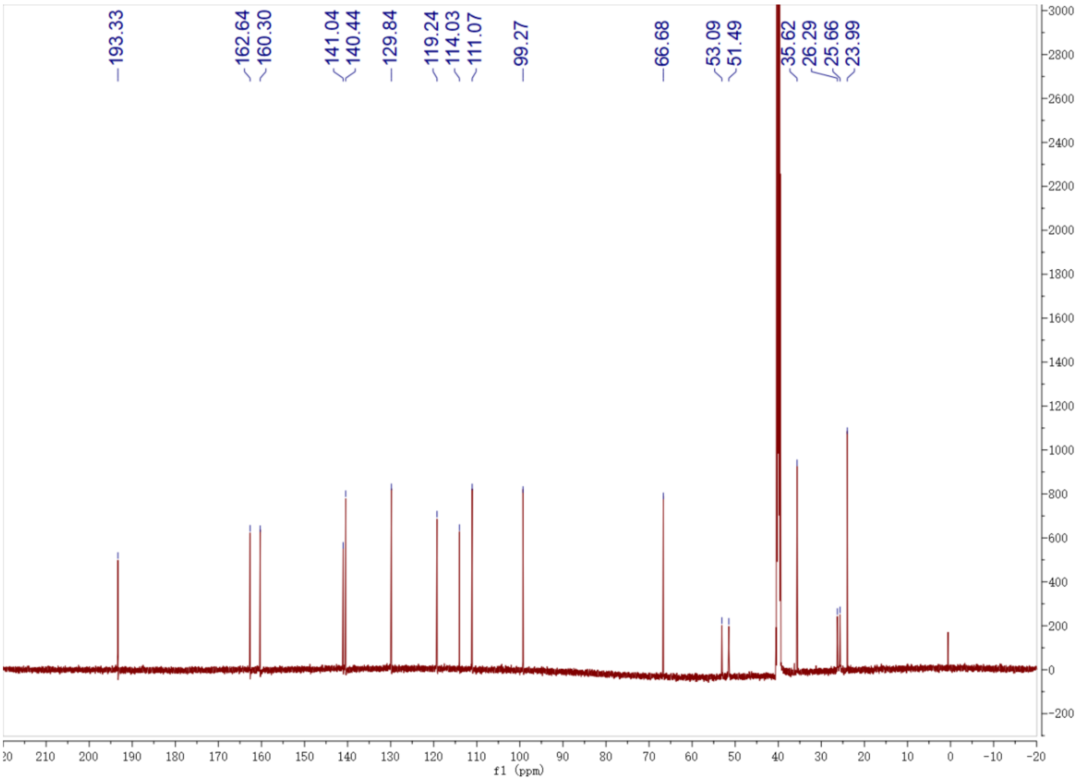

HRMS (ESI) spectrum of compound **4a**.

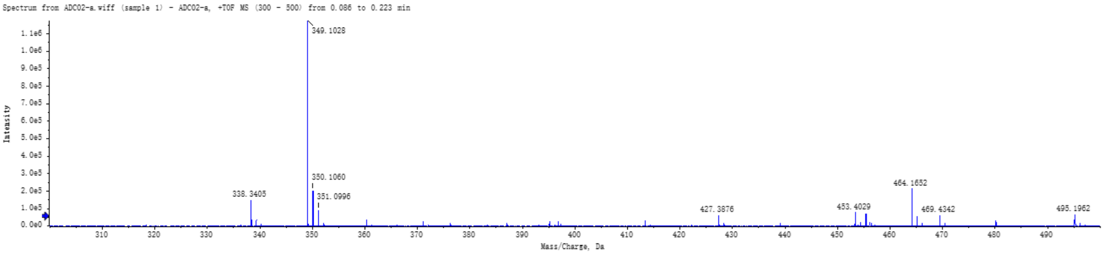

[1] H NMR spectrum of compound **4b**

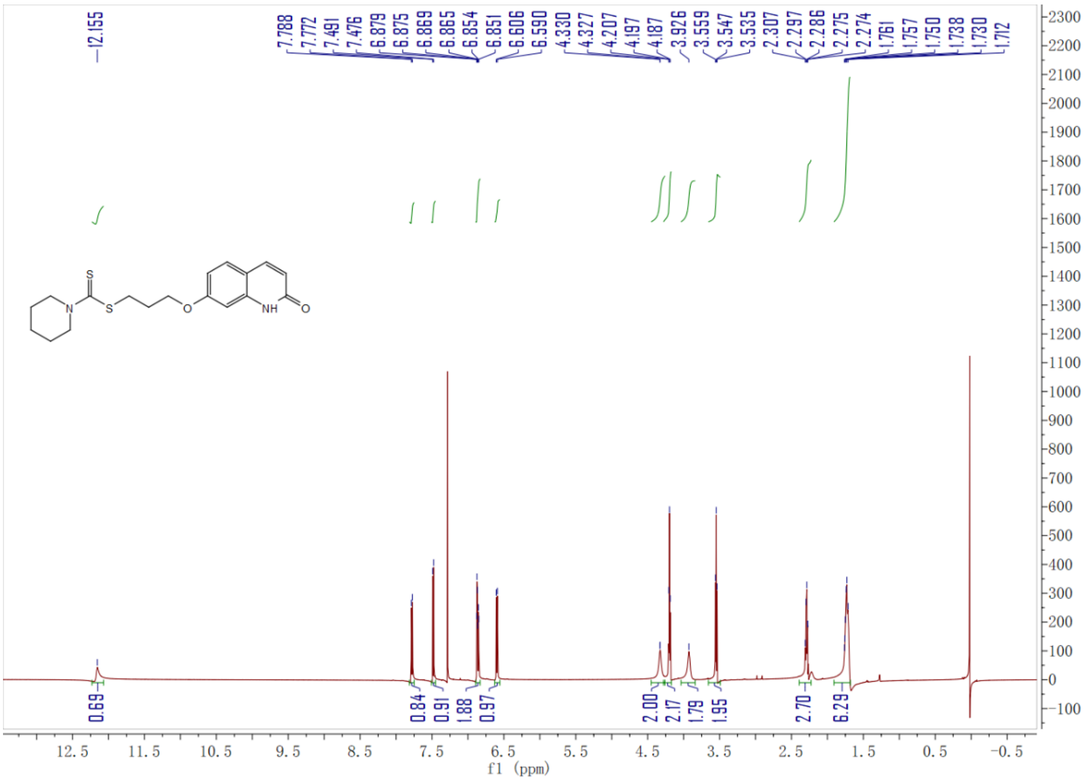

[13] C NMR spectrum of compound **4b**

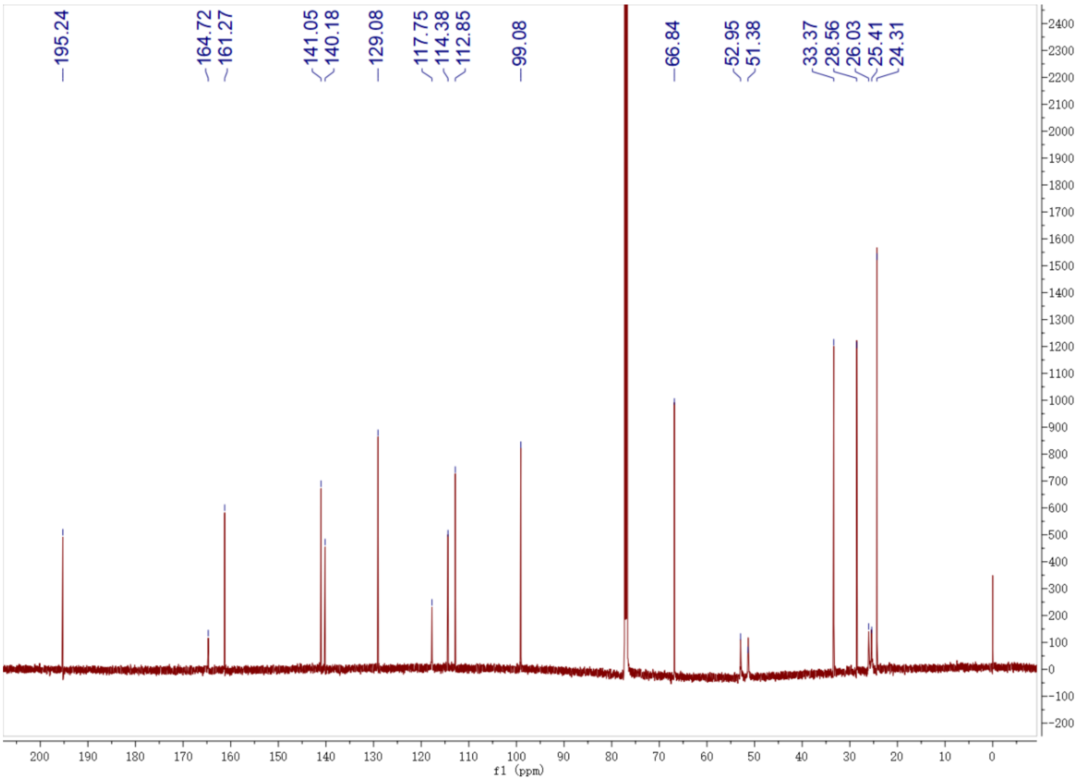

HRMS (ESI) spectrum of compound **4b**.

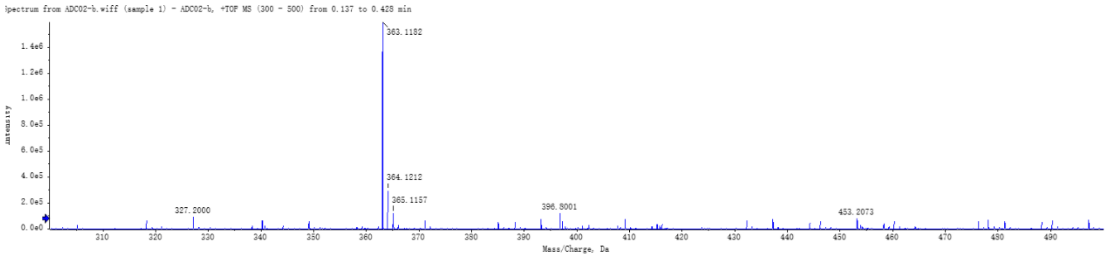

[1] H NMR spectrum of compound **4c**

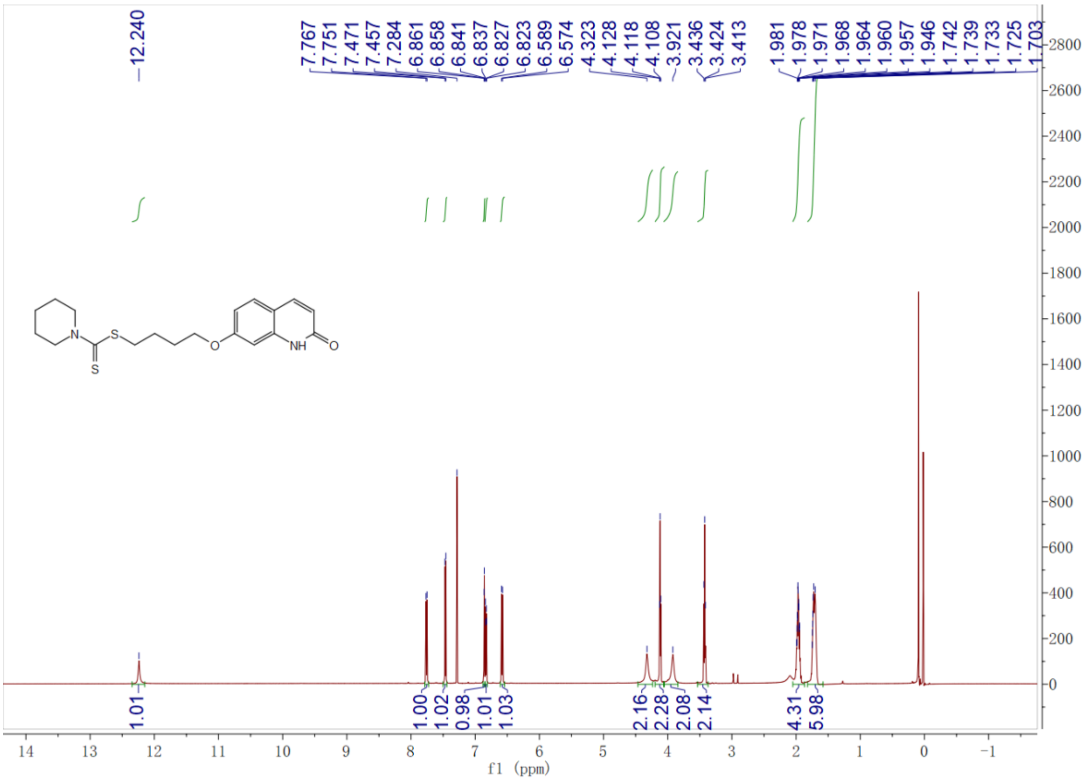

[13] C NMR spectrum of compound **4c**

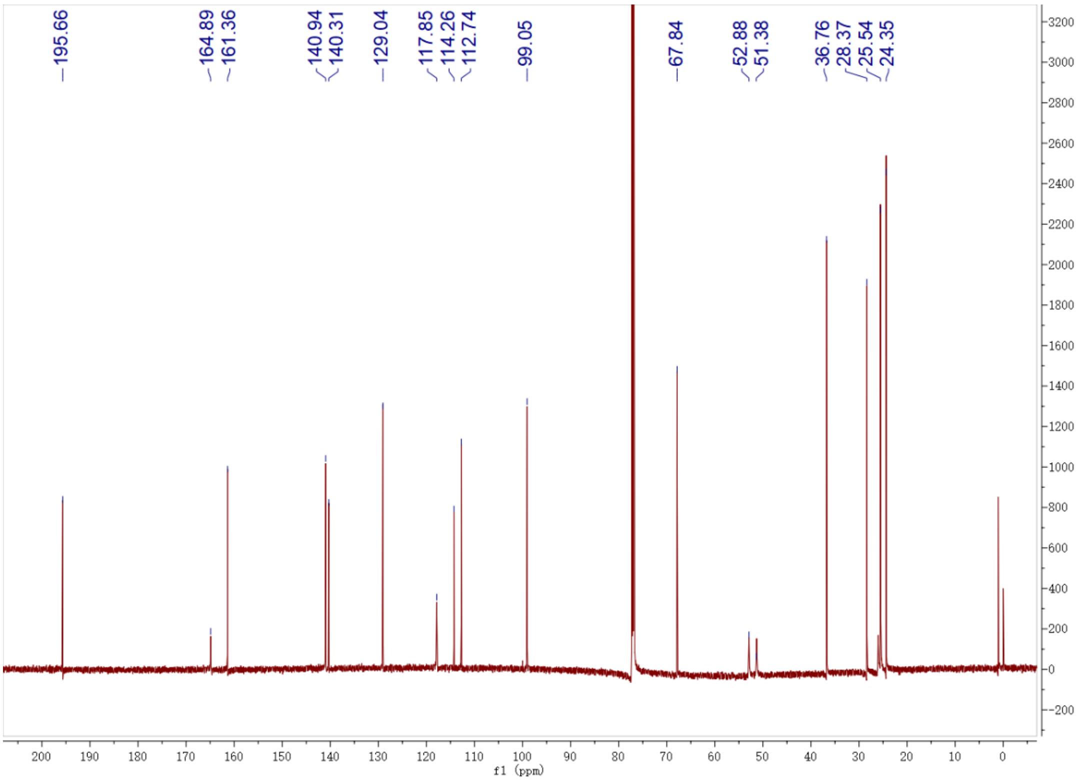

HRMS (ESI) spectrum of compound **4c**.

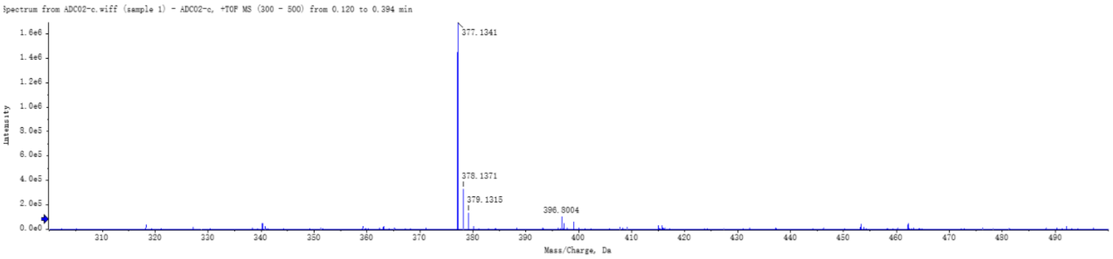

^{1} H NMR spectrum of compound **4d**

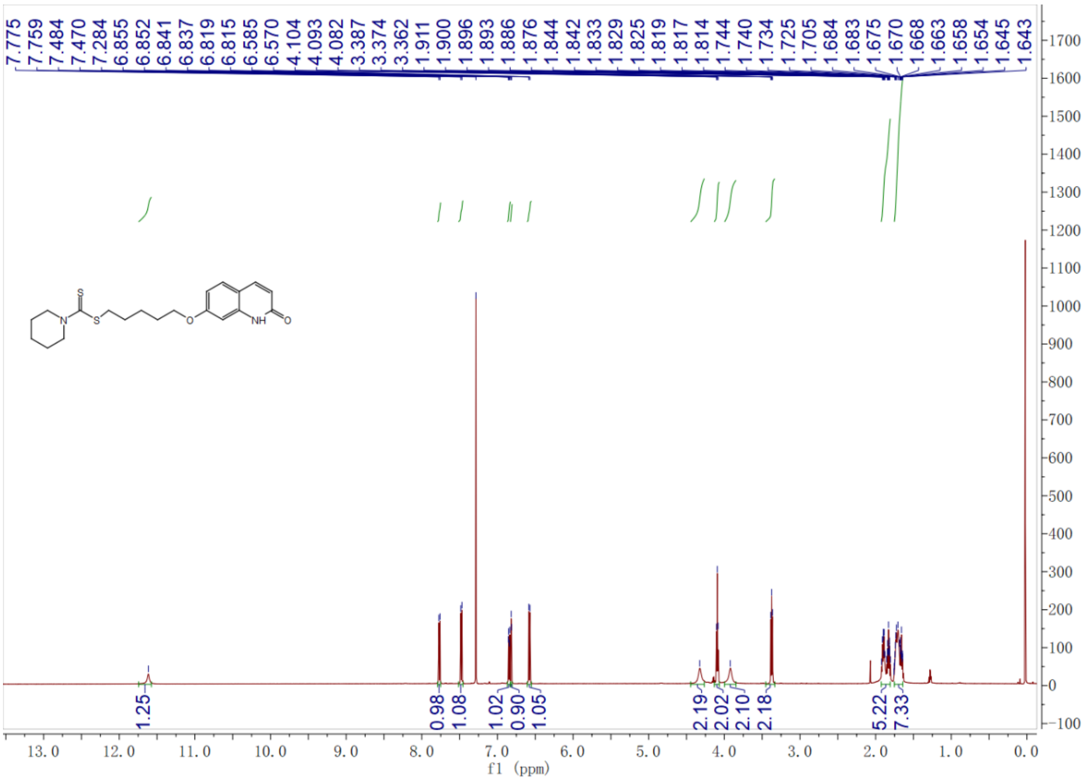

^{13} C NMR spectrum of compound **4d**

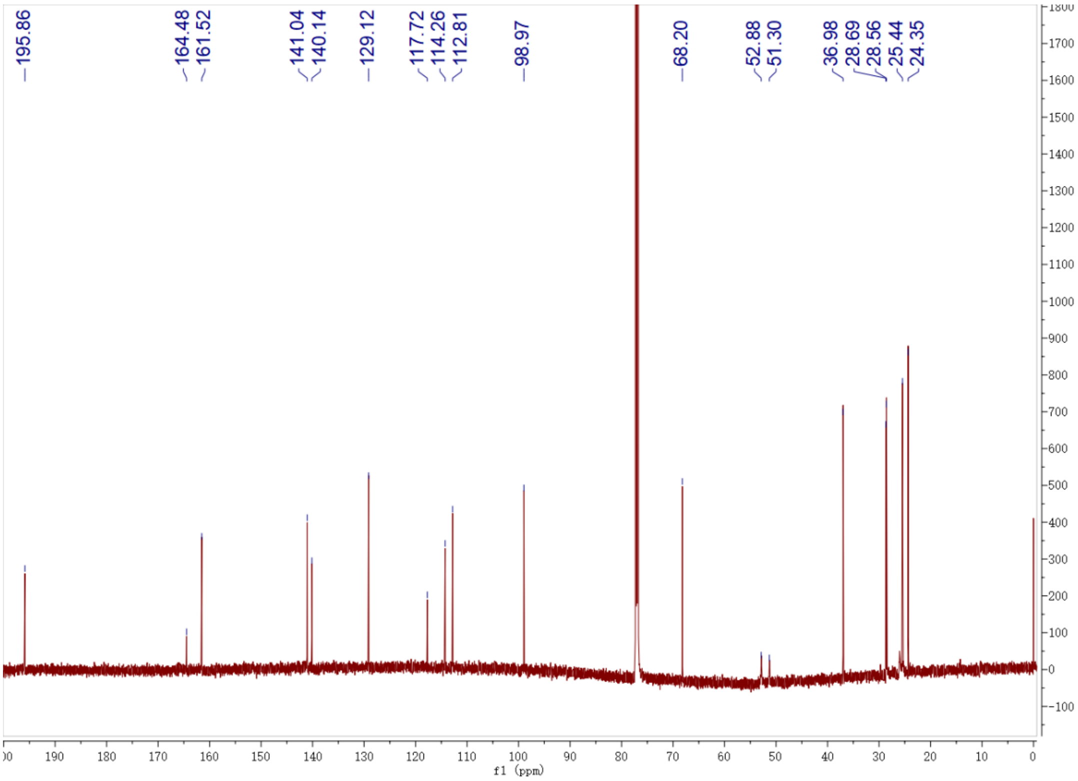

HRMS (ESI) spectrum of compound **4d**.

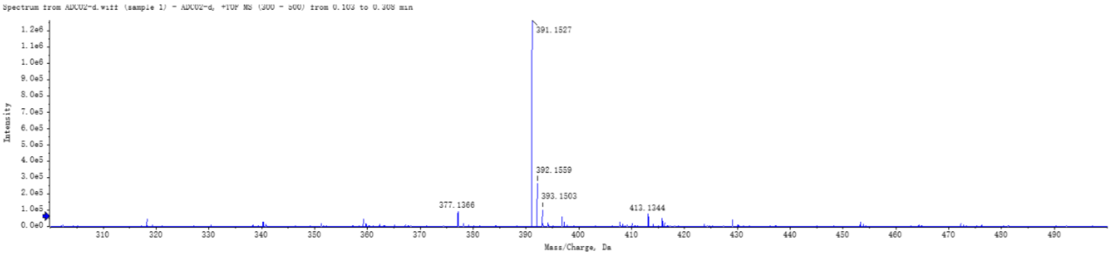

[1] H NMR spectrum of compound **4e**

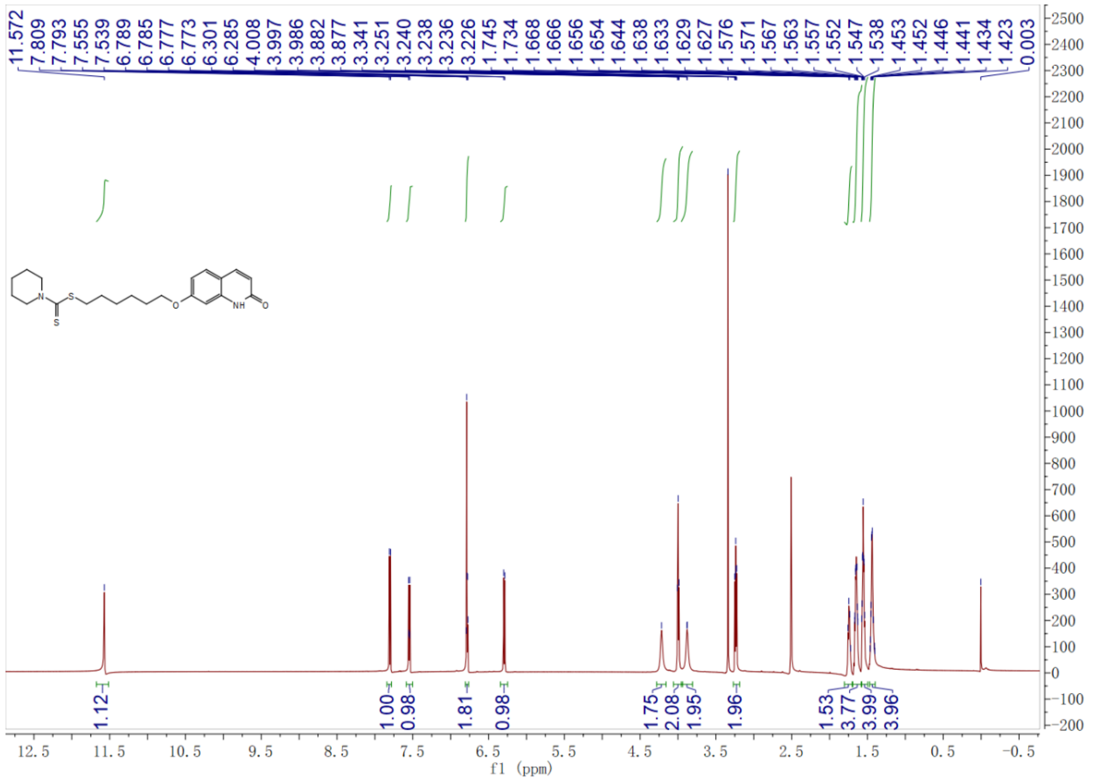

[13] C NMR spectrum of compound **4e**

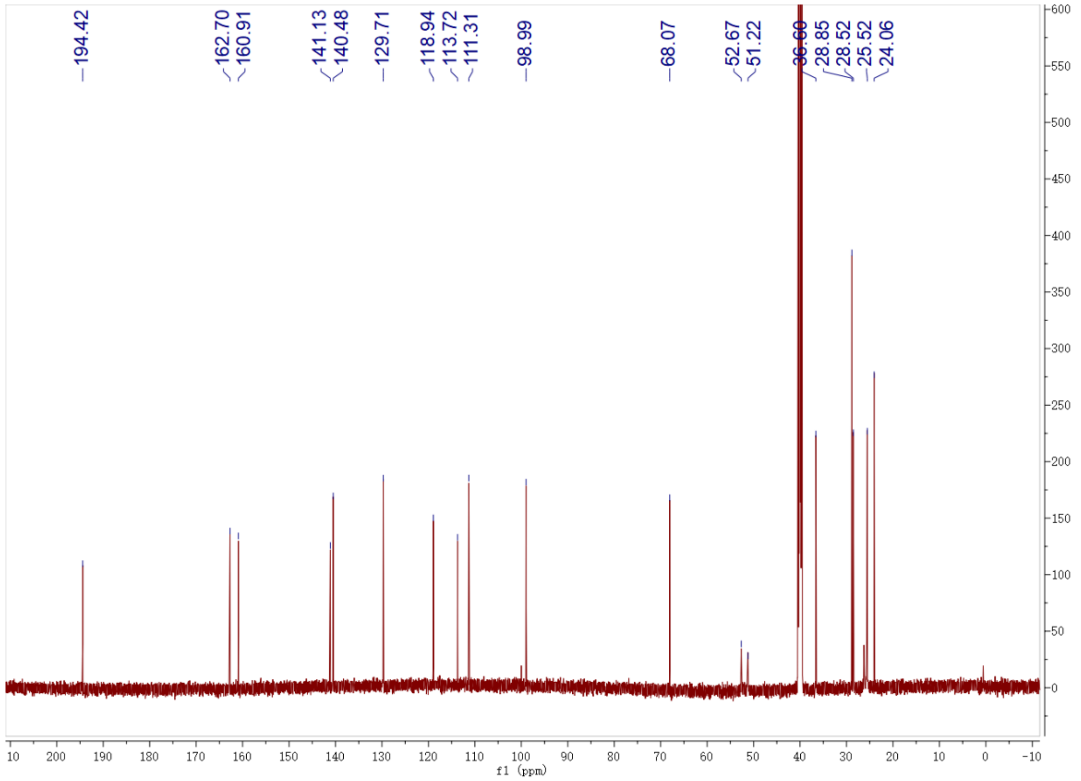

HRMS (ESI) spectrum of compound **4e**.

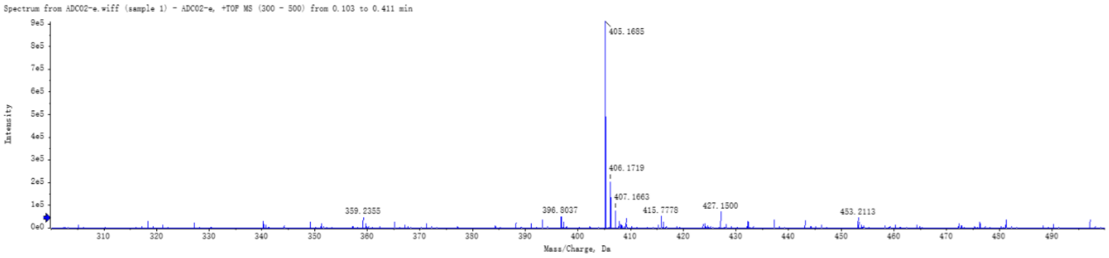

[1] H NMR spectrum of compound **4f**

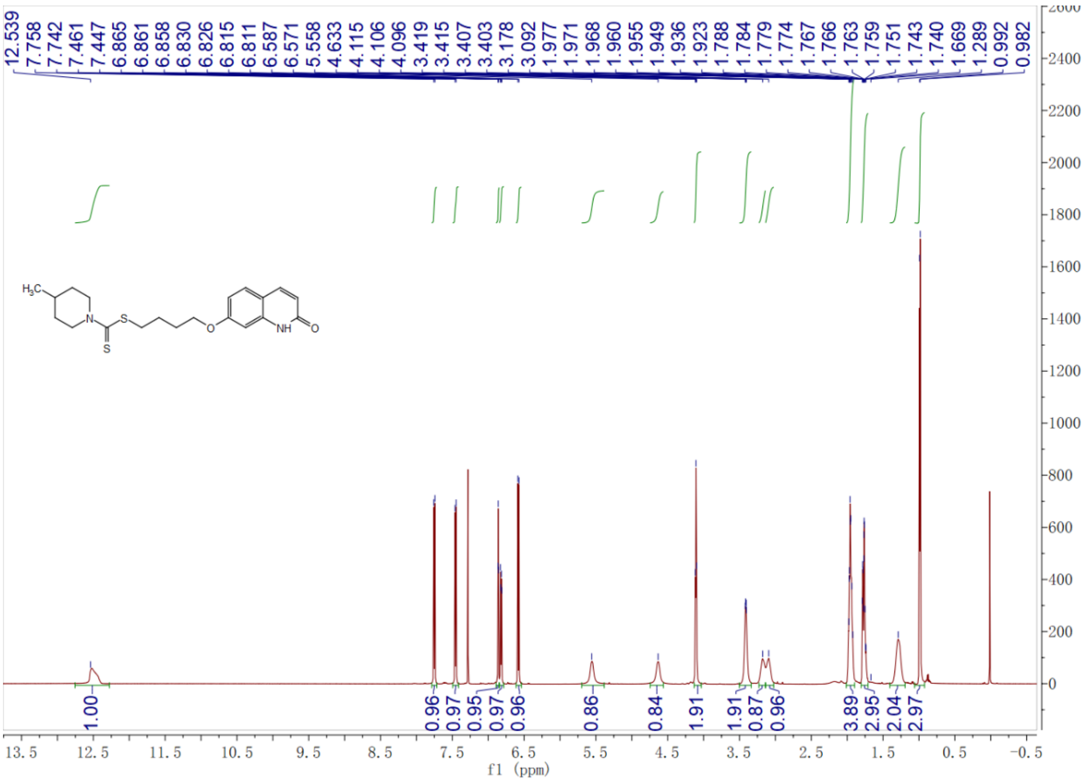

[13] C NMR spectrum of compound **4f**

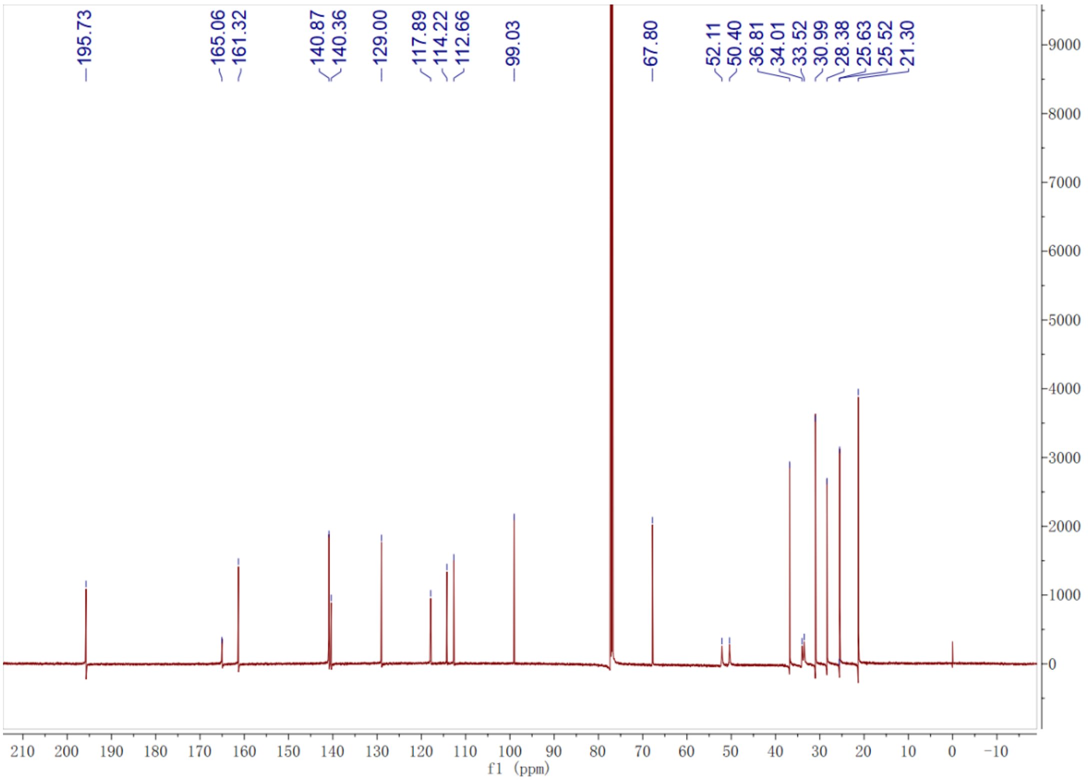

HRMS (ESI) spectrum of compound **4f**.

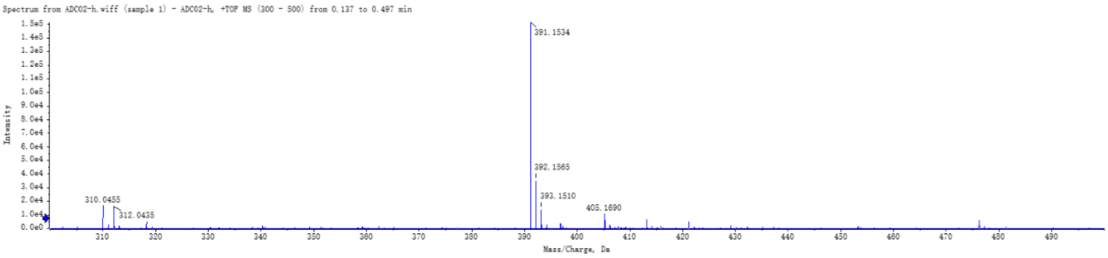

[1] H NMR spectrum of compound **4g**

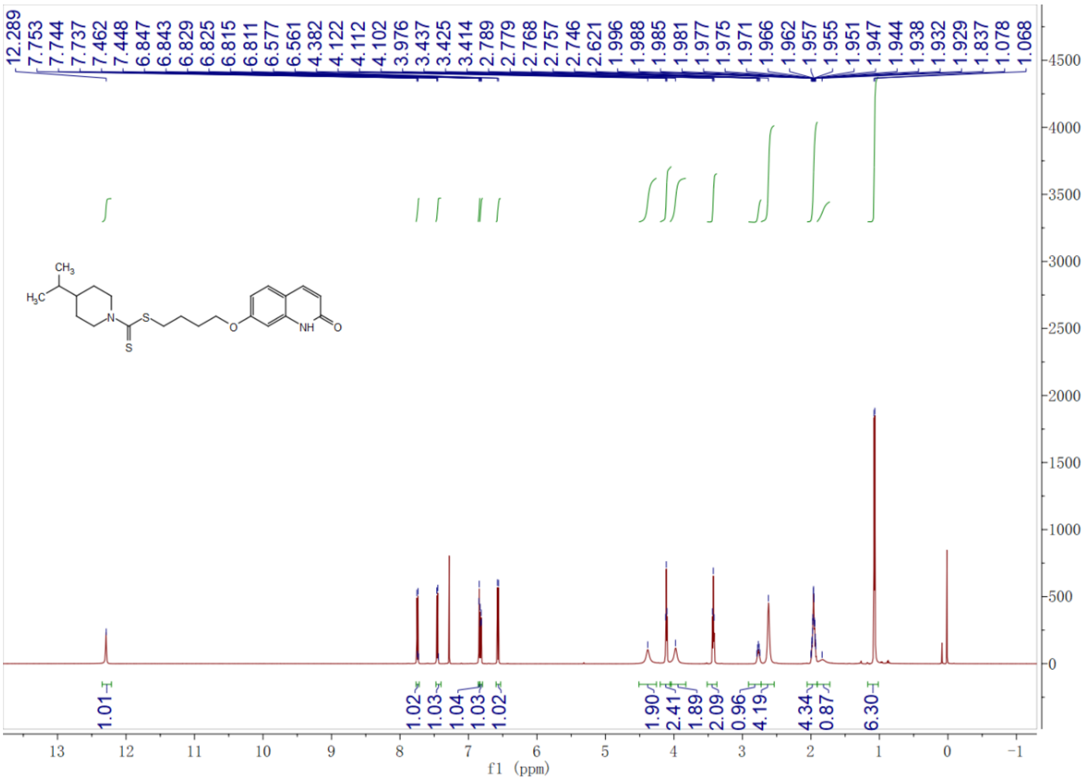

[13] C NMR spectrum of compound **4g**

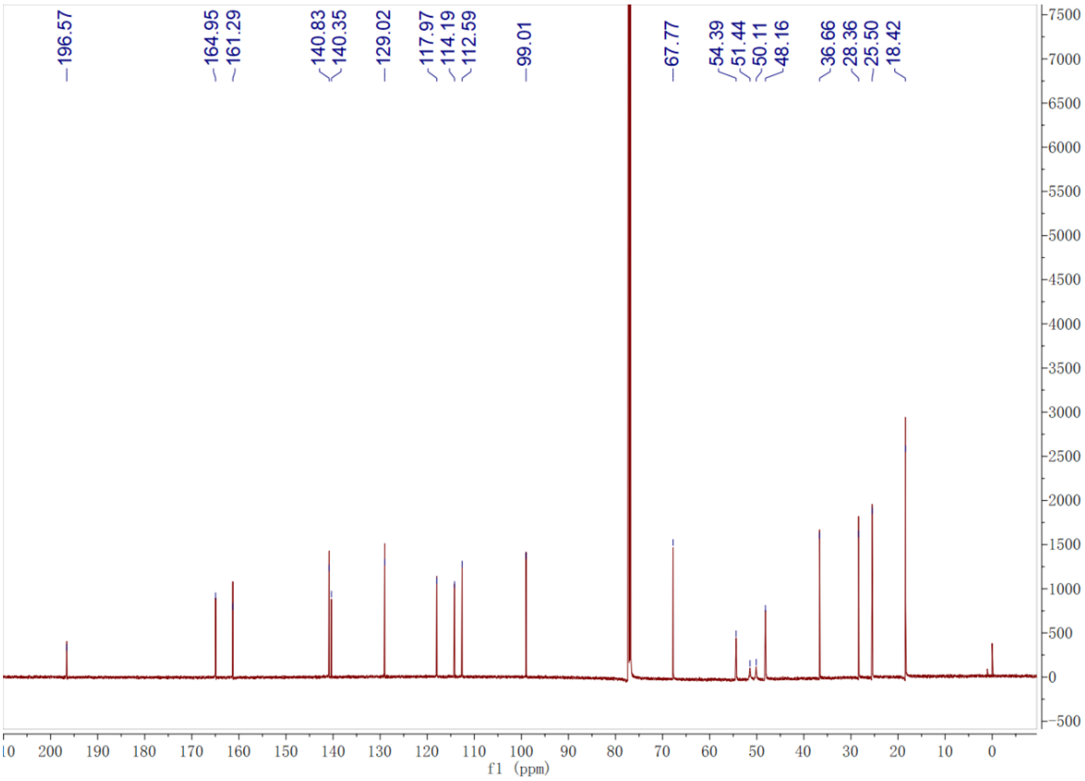

HRMS (ESI) spectrum of compound **4g**.

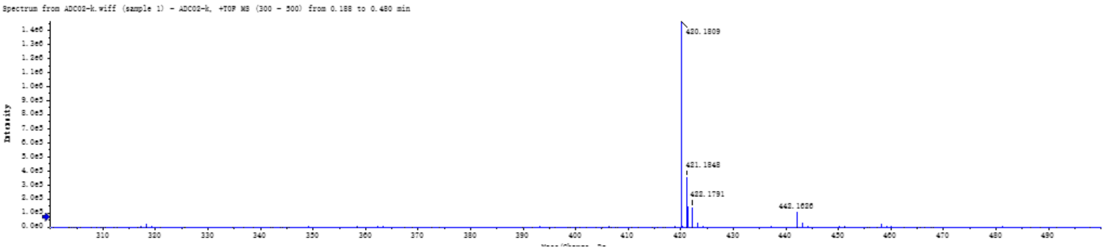

[1] H NMR spectrum of compound **4h**

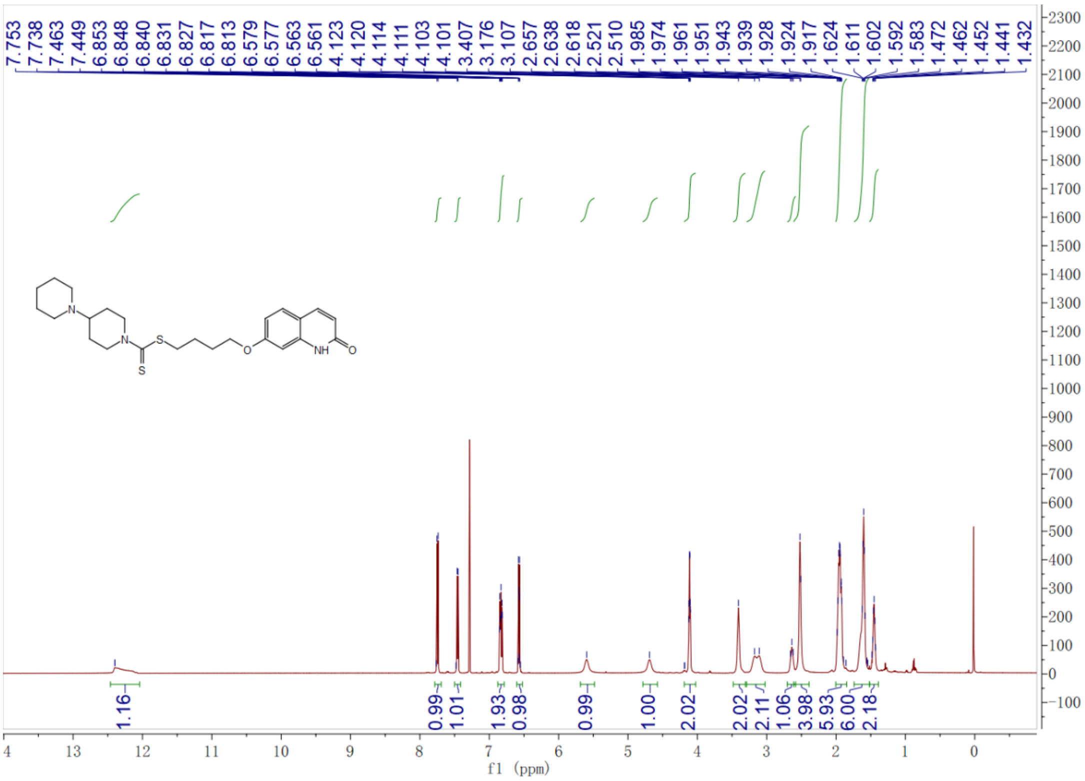

[13] C NMR spectrum of compound **4h**

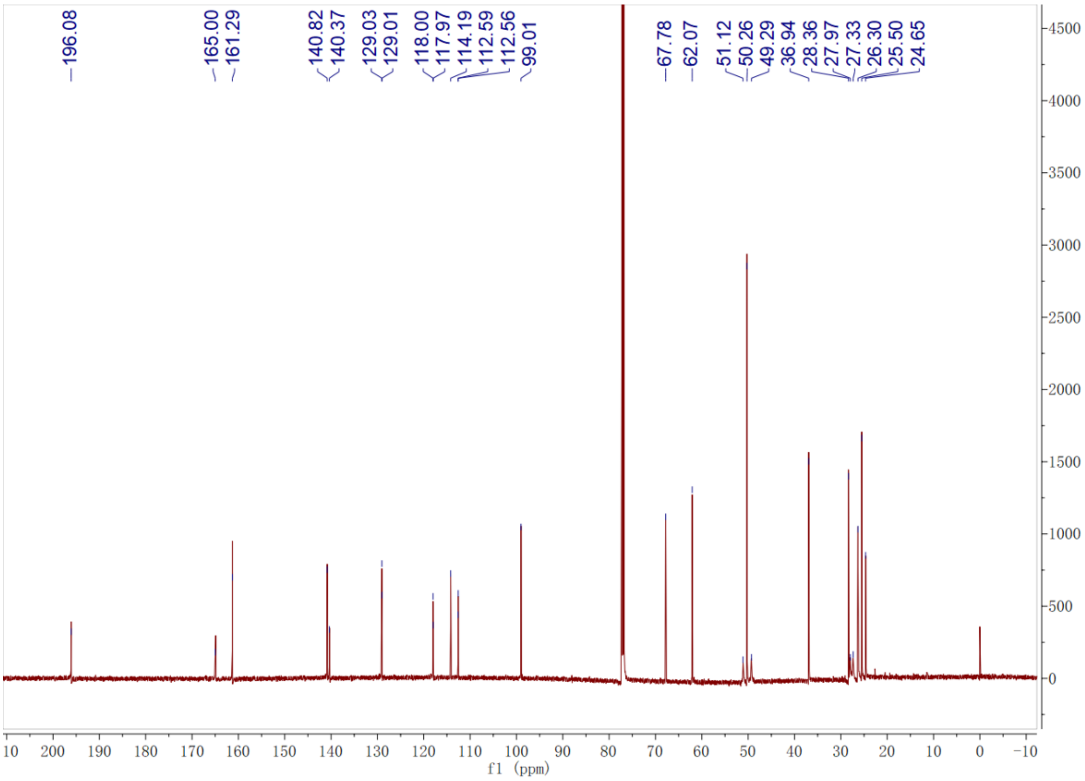

HRMS (ESI) spectrum of compound **4h**.

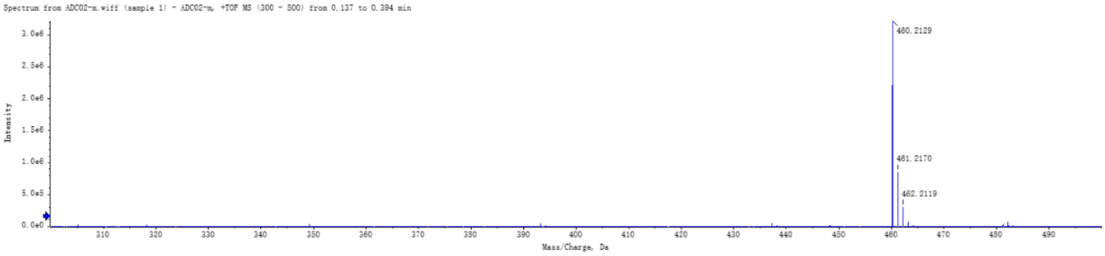

[1] H NMR spectrum of compound **4i**

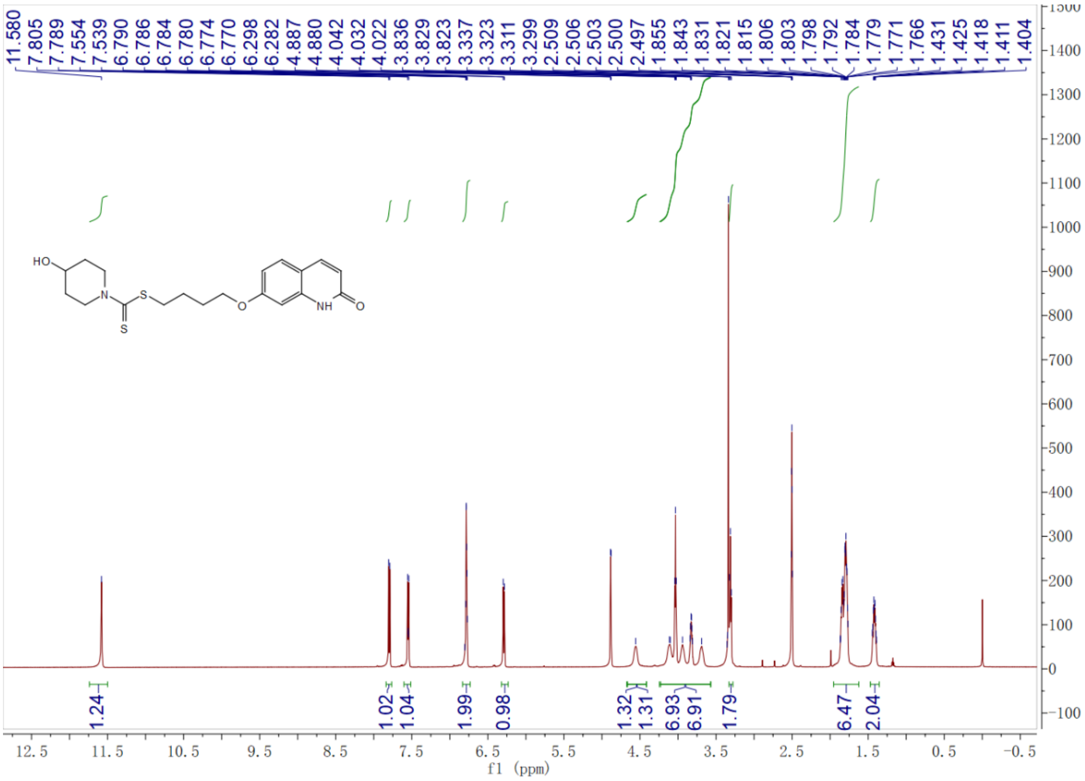

[13] C NMR spectrum of compound **4i**

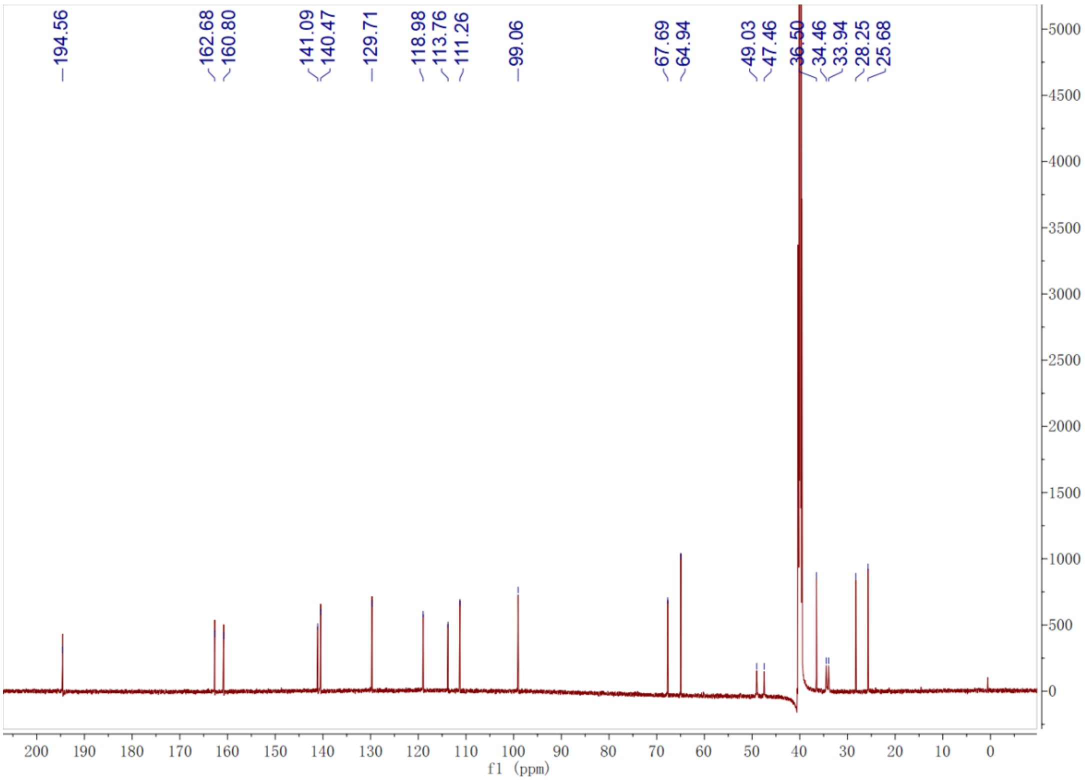

HRMS (ESI) spectrum of compound **4i**.

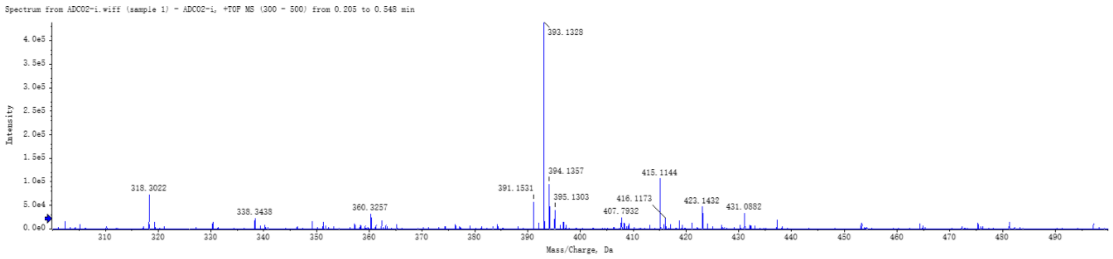

[1] H NMR spectrum of compound **4j**

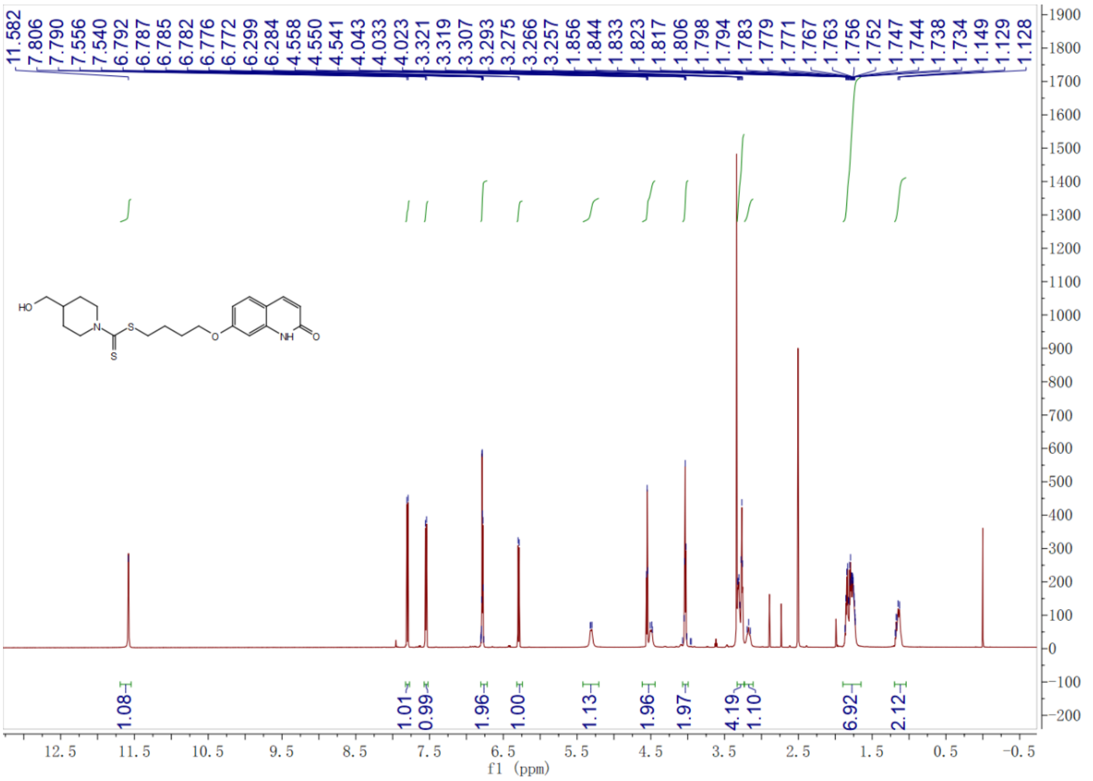

^{13}C NMR spectrum of compound **4j**

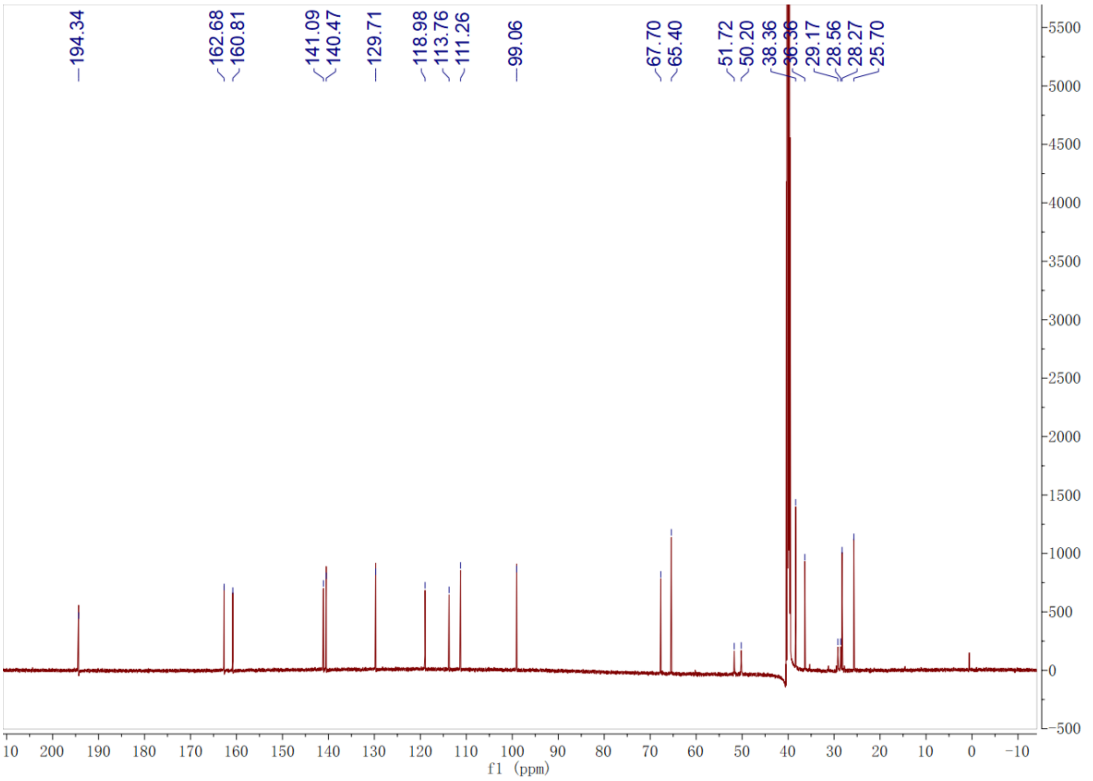

HRMS (ESI) spectrum of compound **4j**.

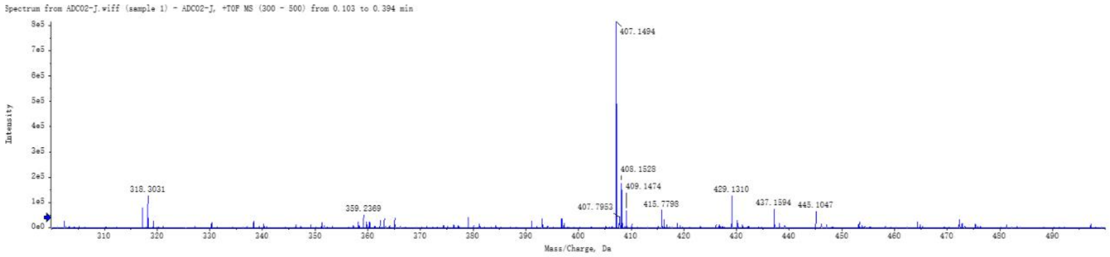

[1] H NMR spectrum of compound **4k**

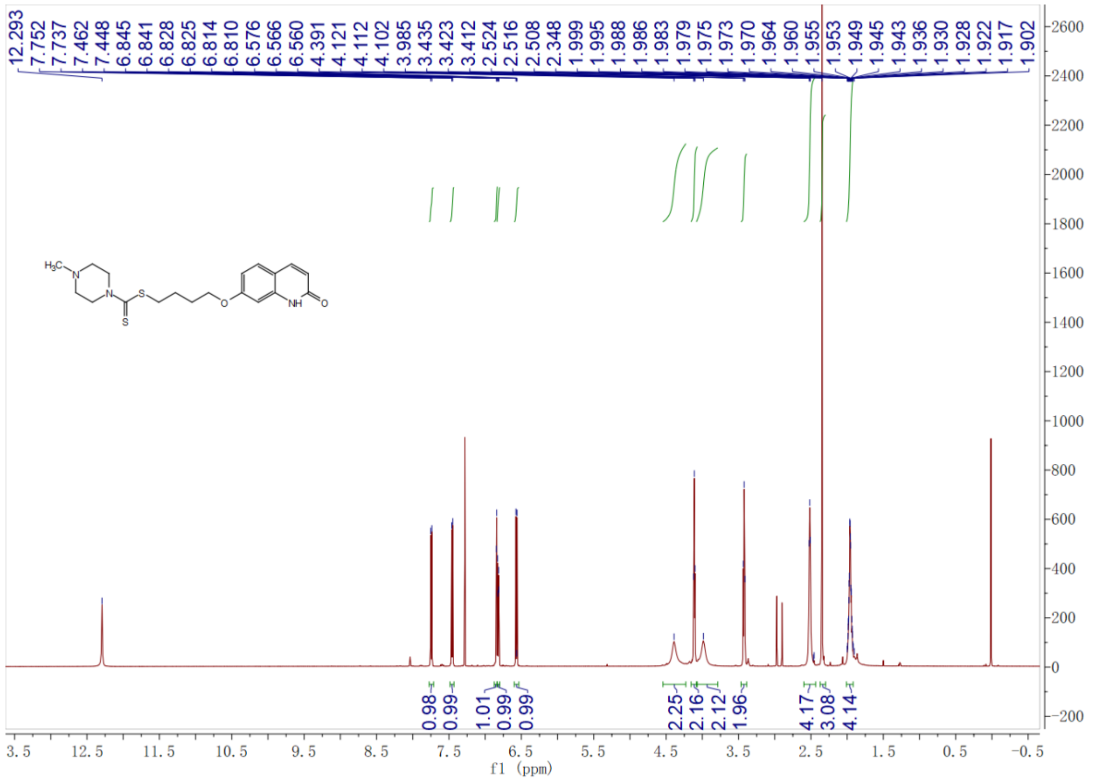

^{13}C NMR spectrum of compound **4k**

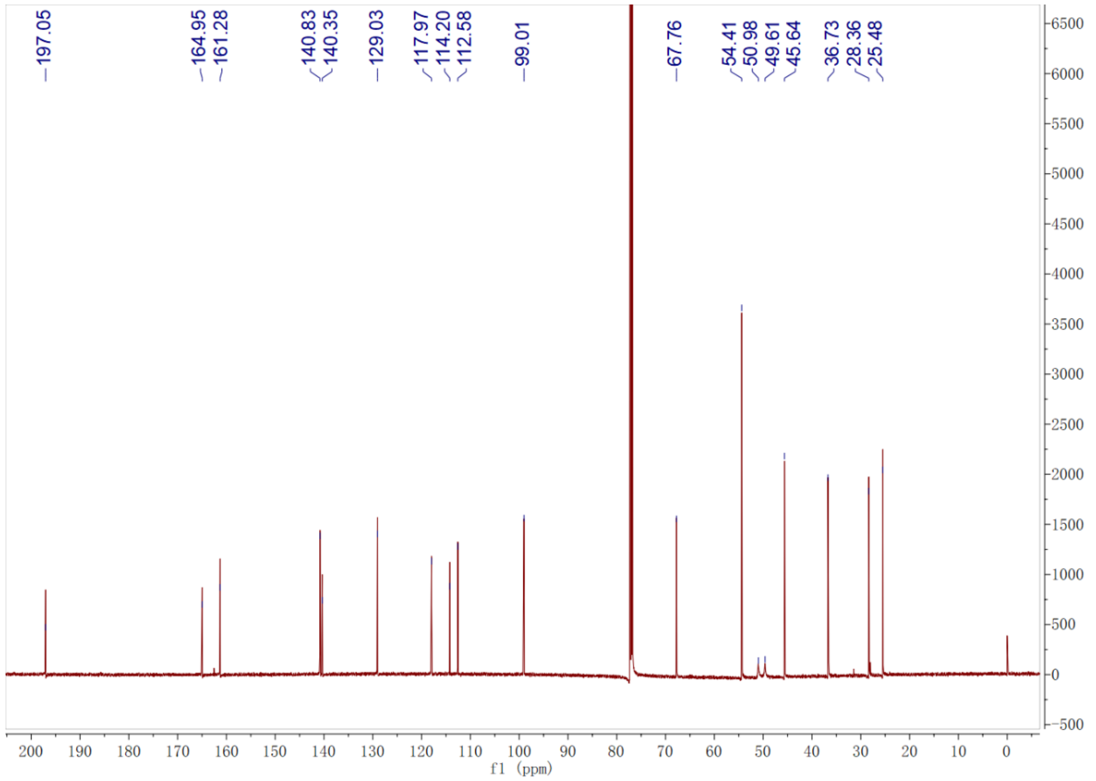

HRMS (ESI) spectrum of compound **4k**.

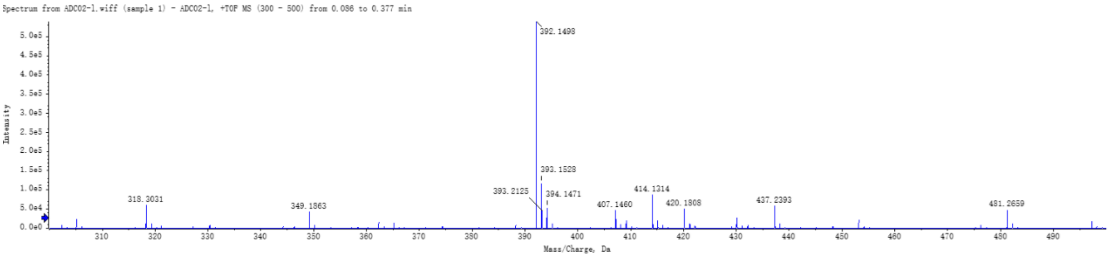

[1] H NMR spectrum of compound **4l**

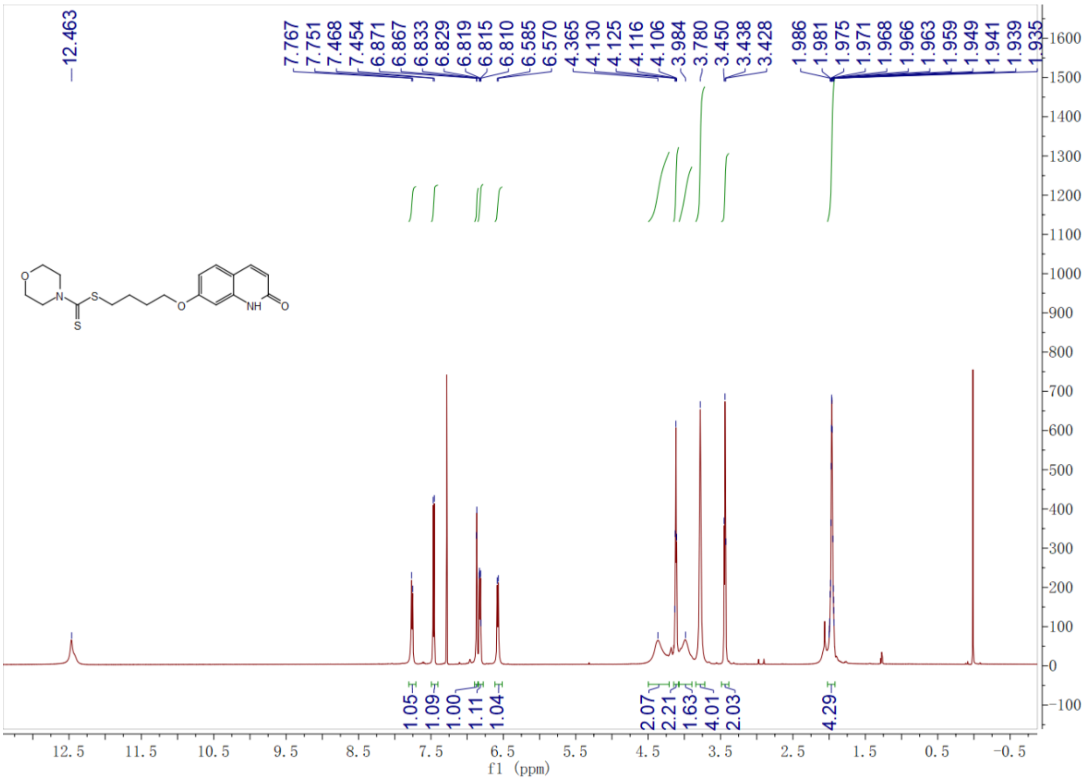

^{13}C NMR spectrum of compound **4l**

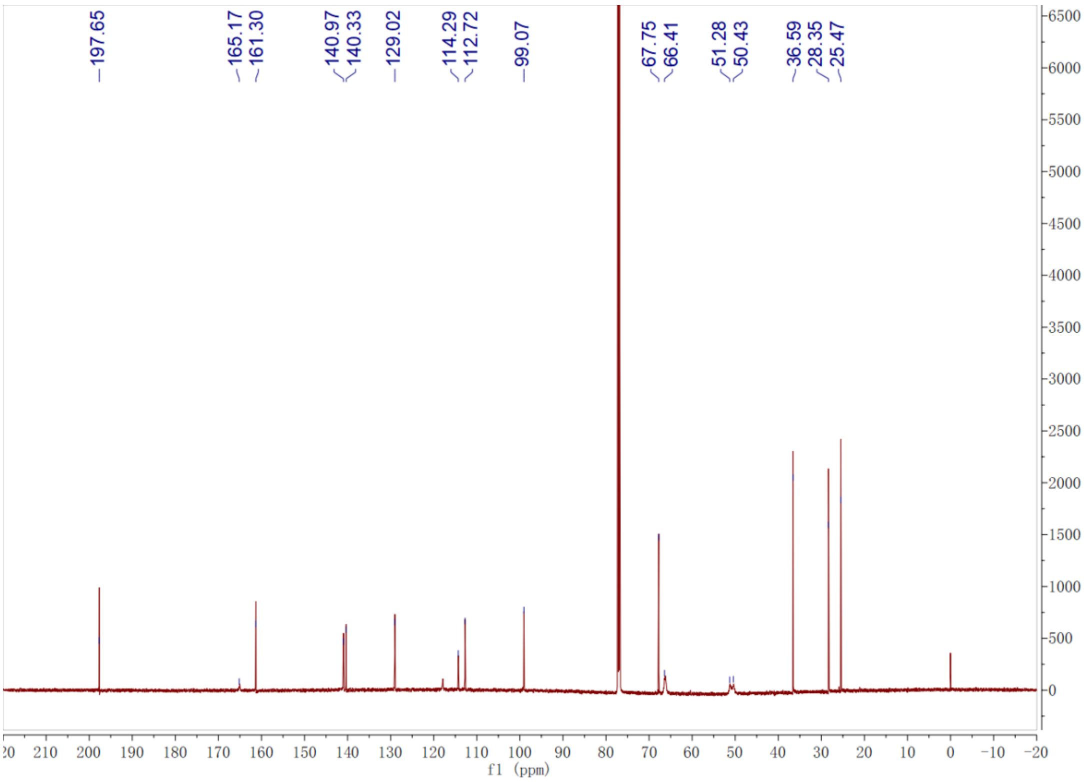

HRMS (ESI) spectrum of compound **4l**.

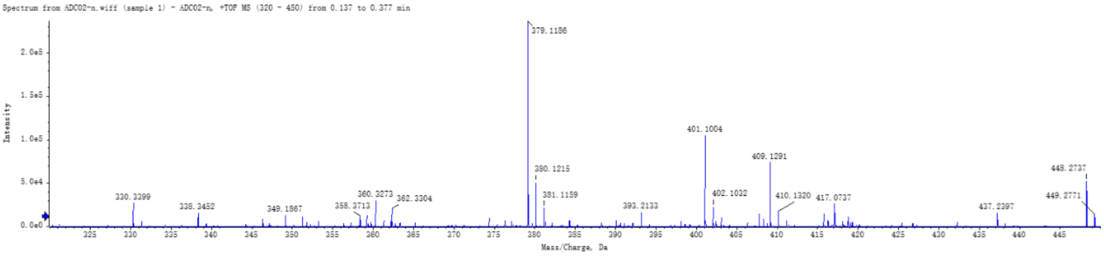

[1] H NMR spectrum of compound **4m**

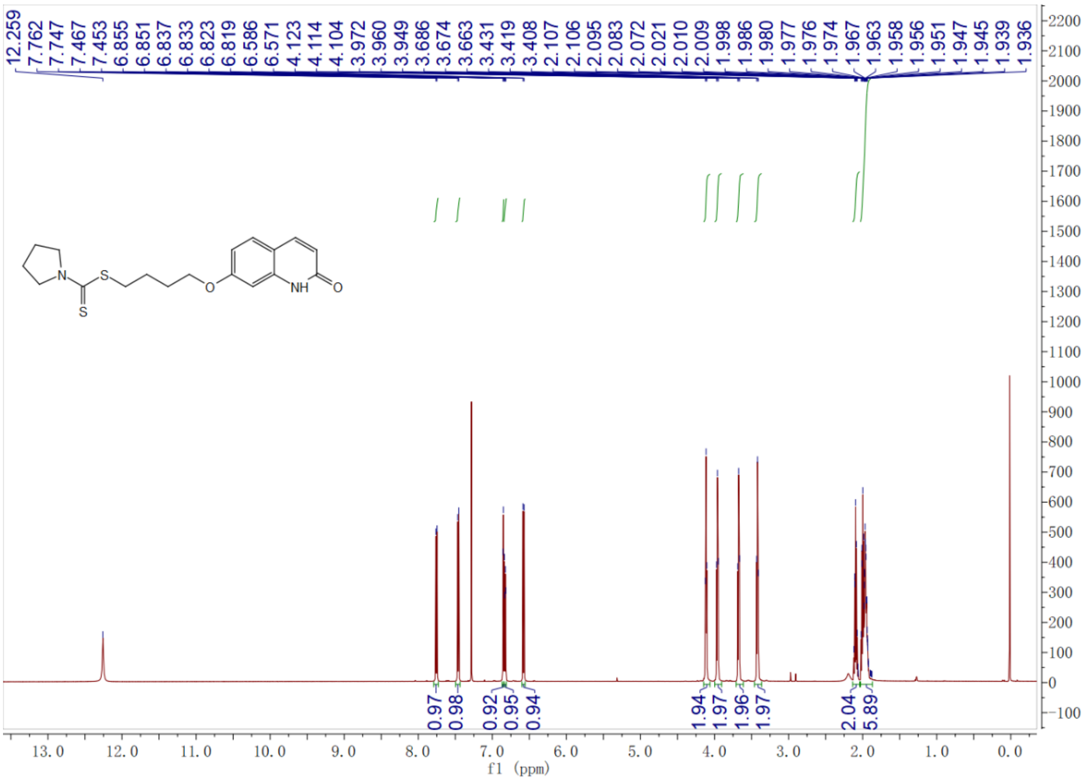

^{13}C NMR spectrum of compound **4m**

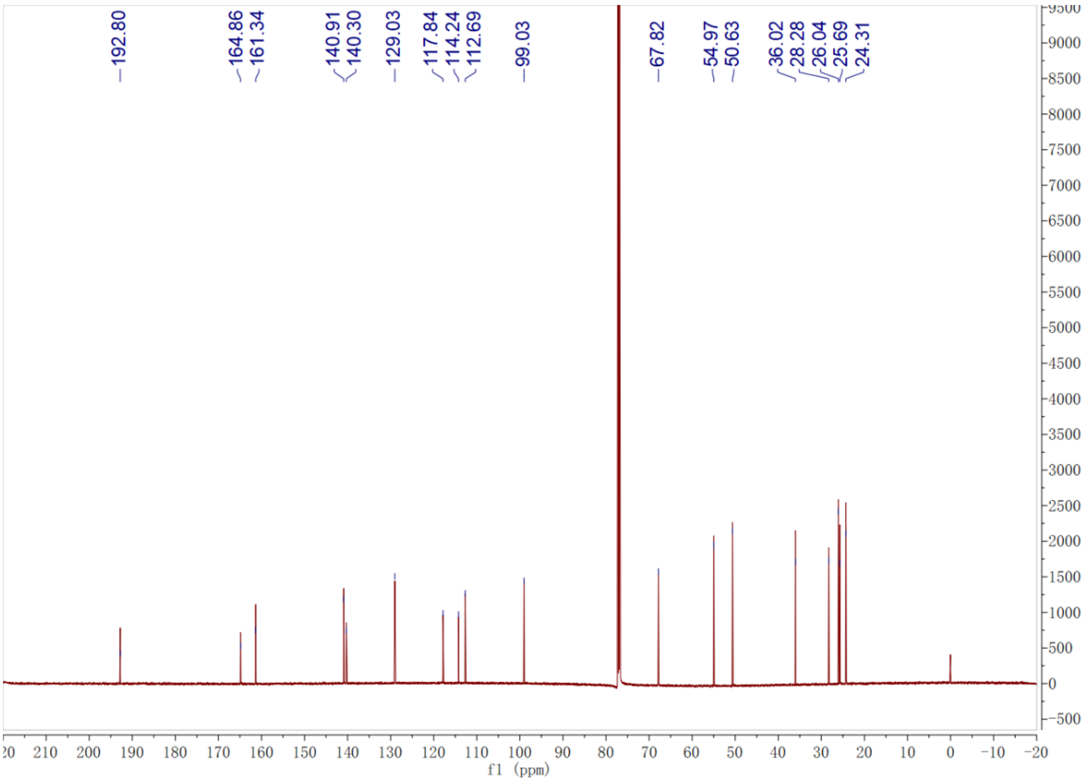

HRMS (ESI) spectrum of compound **4m**

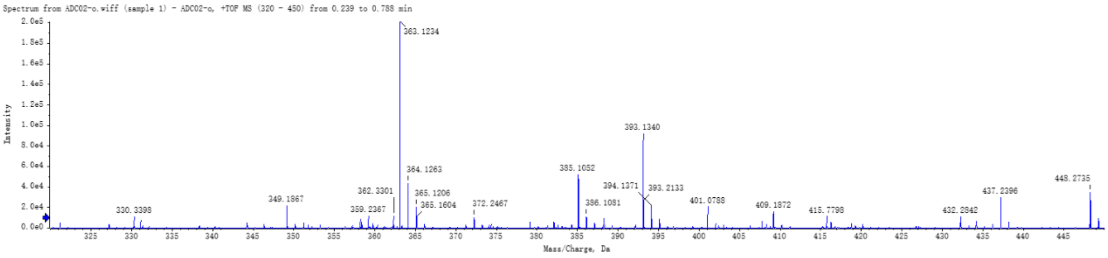

[1] H NMR spectrum of compound **9a**

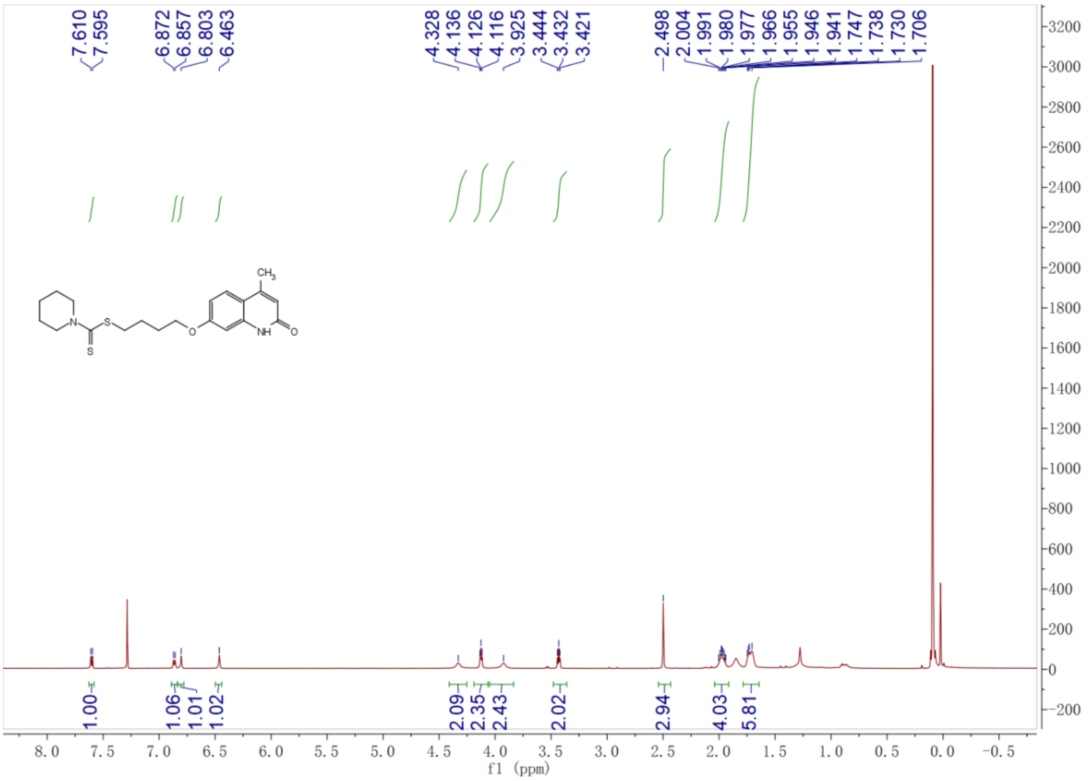

^{13}C NMR spectrum of compound **9a**

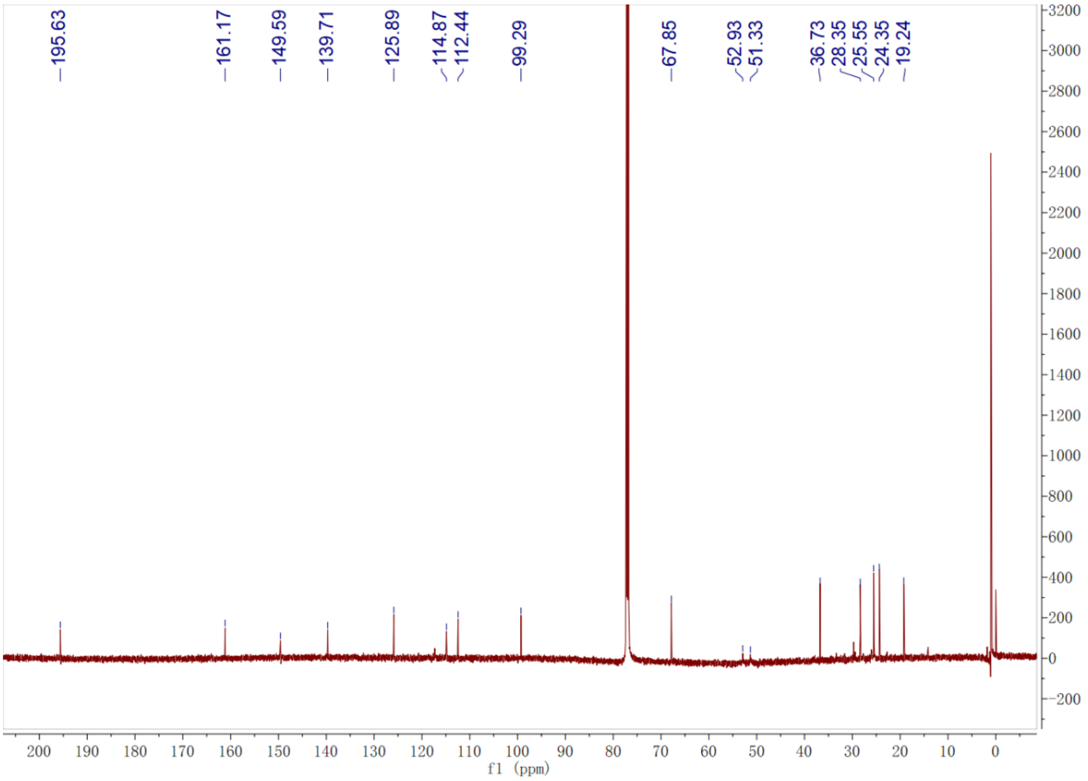

HRMS (ESI) spectrum of compound **9a**

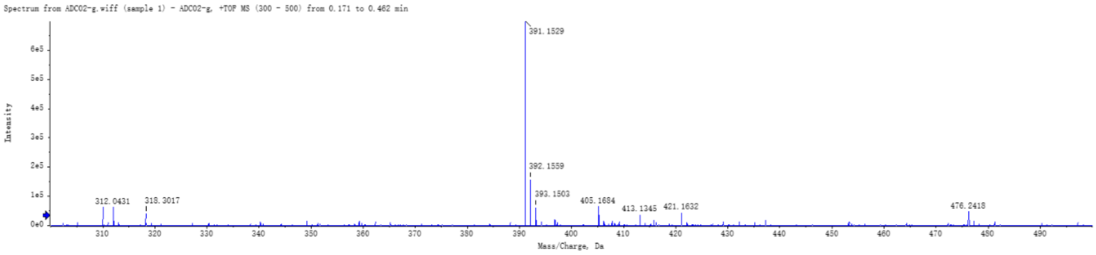

[1] H NMR spectrum of compound **9b**

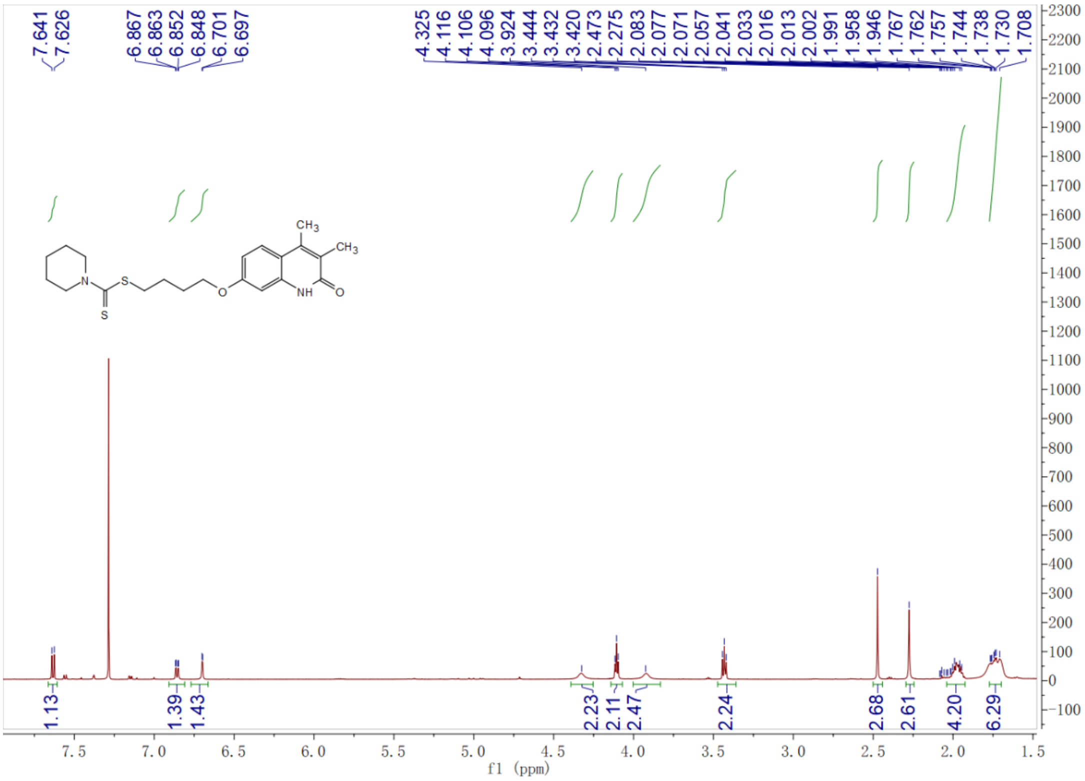

^{13}C NMR spectrum of compound **9b**

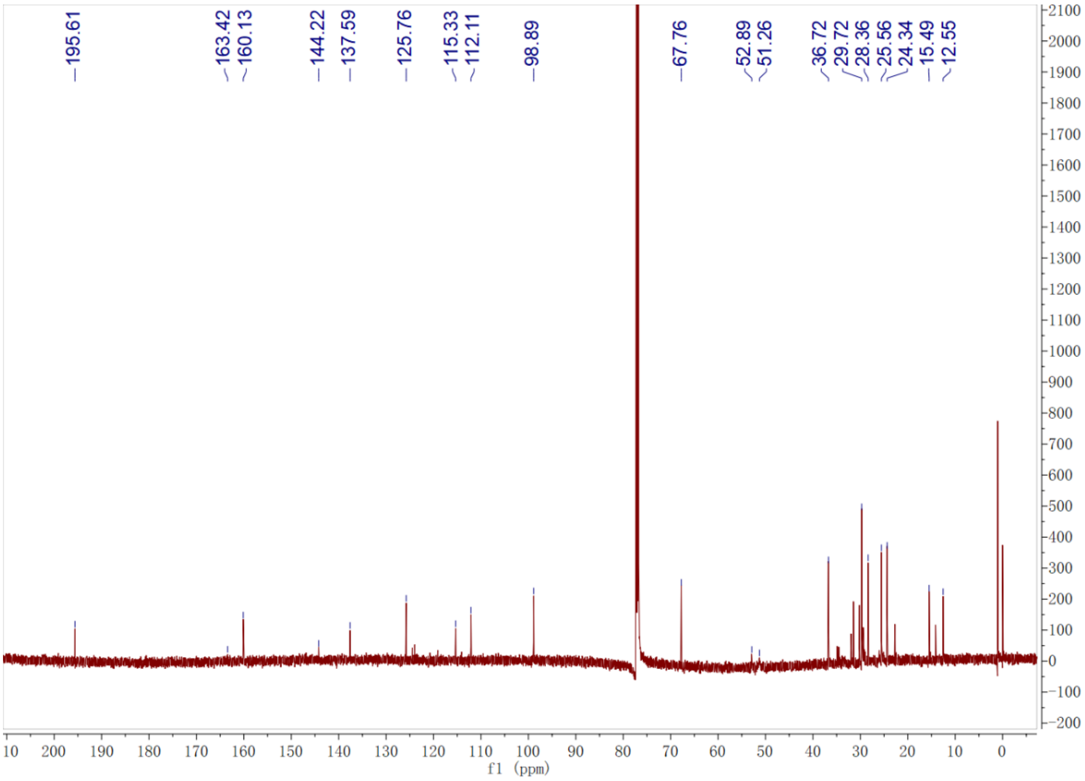

HRMS (ESI) spectrum of compound **9b**

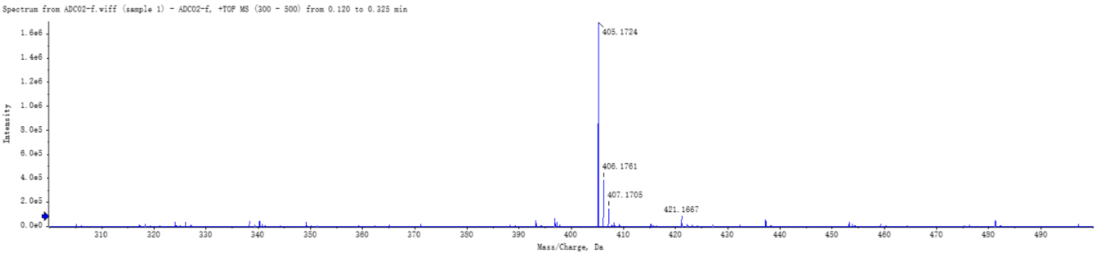

Table S1 S value of the compounds **4a-m** and **9a-b** in active site of AChE.

**Compound**	**S value**	
	*h*AChE(4EY7)	*Tc*AChE(2CKM)
**4a**	-6.237	-6.940
**4b**	-7.025	-7.061
**4c**	**-7.863**	**-7.540**
**4d**	-7.739	-7.154
**4e**	-7.384	-7.238
**4f**	-7.350	-7.119
**4g**	-7.027	-7.002
**4h**	-7.000	-7.039
**4i**	-6.105	-5.580
**4j**	-6.557	-5.719
**4k**	-6.399	-4.353
**4l**	-6.054	-6.091
**4m**	-6.605	-5.750
**9a**	-7.631	-7.155
**9b**	-7.851	-7.468
**donepezil**	-8.300	-7.472

Table S2 Docking orientations of compounds **4a-m** and **9a-b** with *h*AChE and *Tc*AChE.

**Compd.**	***h*AChE (4EY7)**	***Tc*AChE(2CKM)**
**4a**	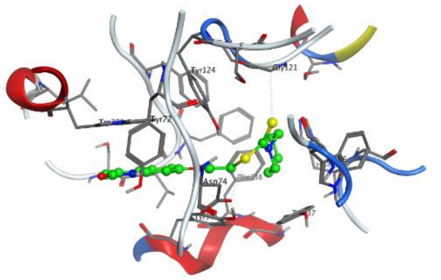	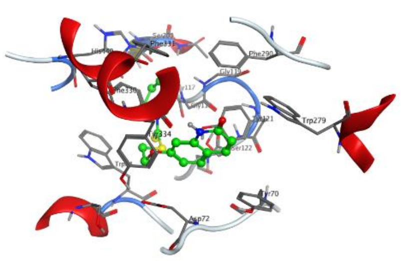
**4b**	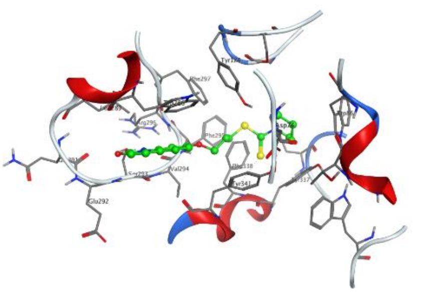	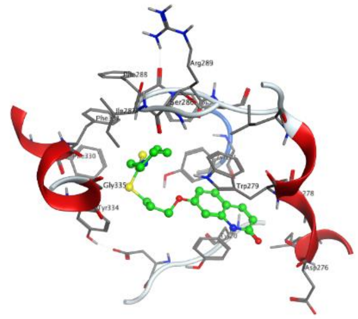
**4c**	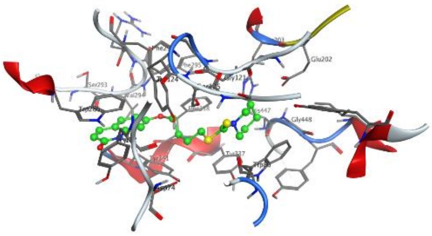	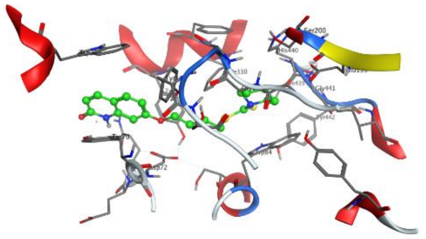
**4d**	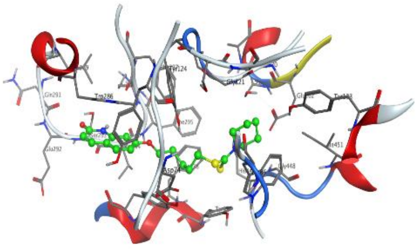	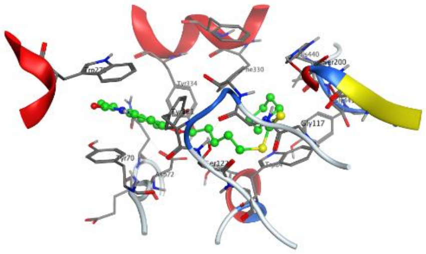
**4e**	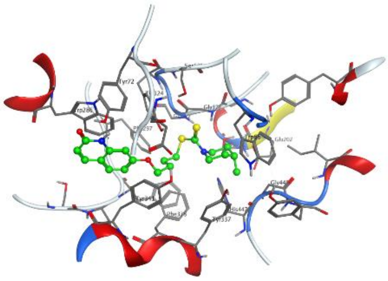	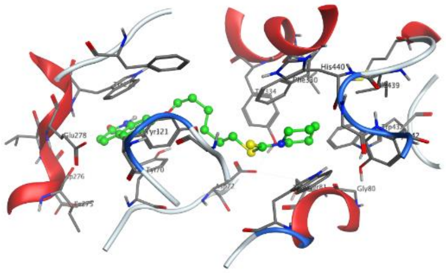

**4f**	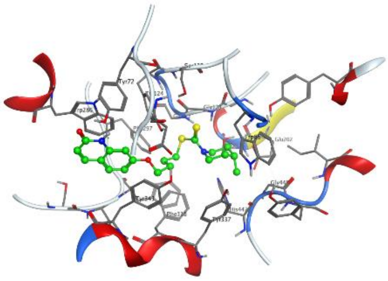	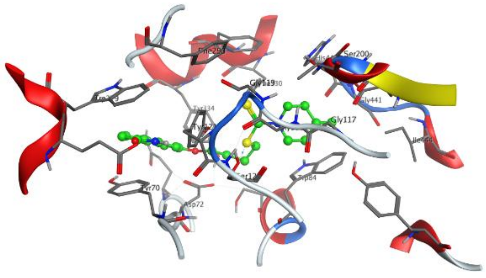
**4g**	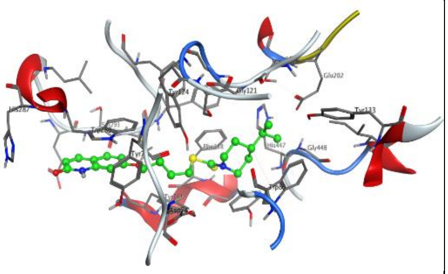	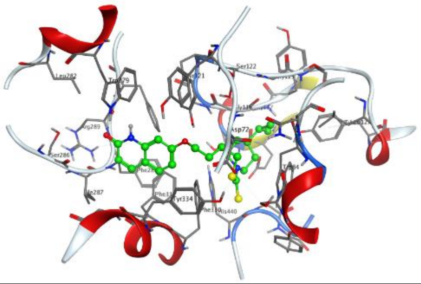
**4h**	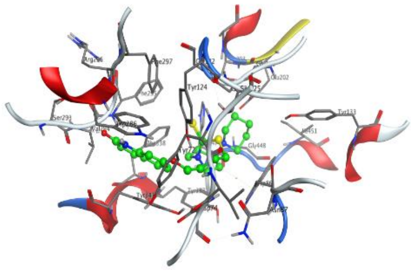	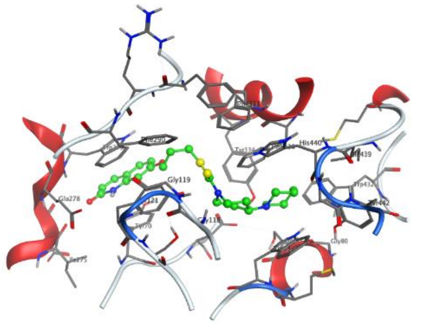
**4i**	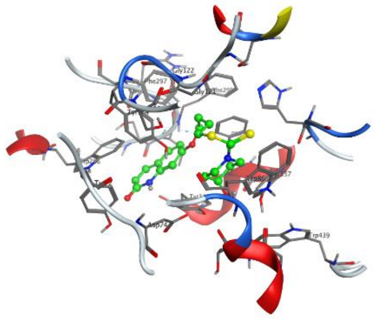	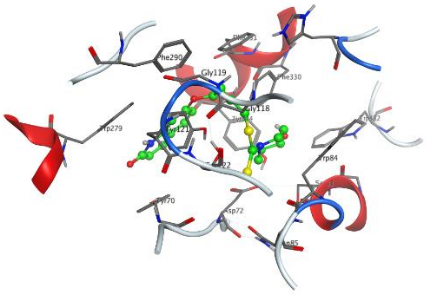
**4j**	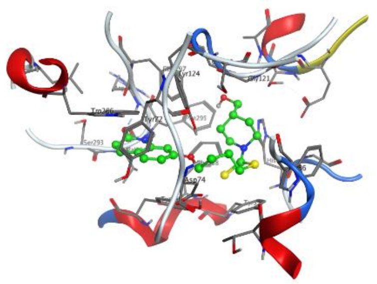	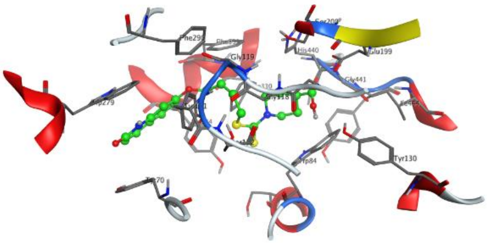

**4k**	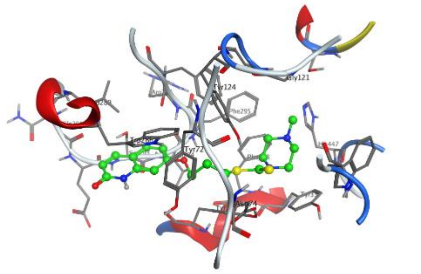	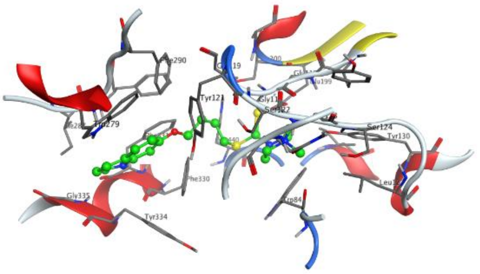
**4l**	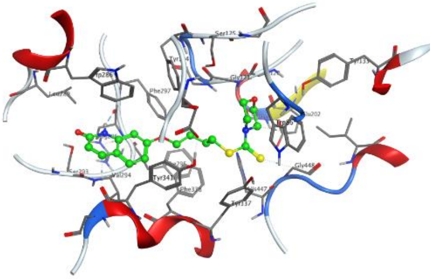	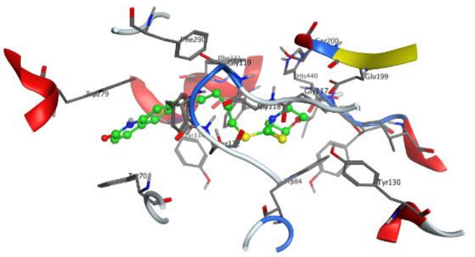
**4m**	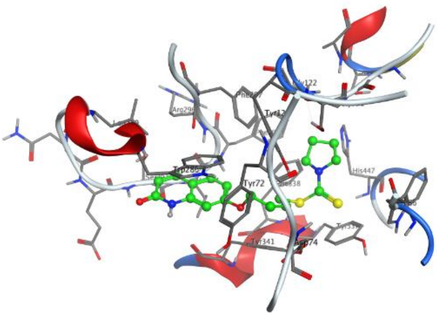	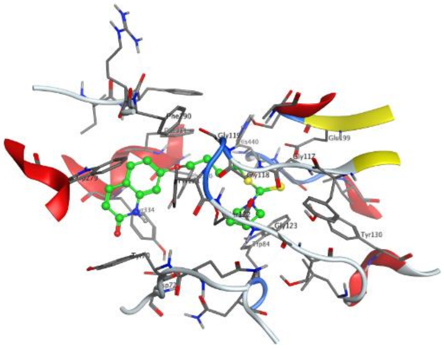
**9a**	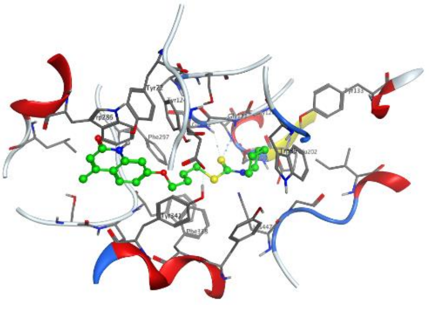	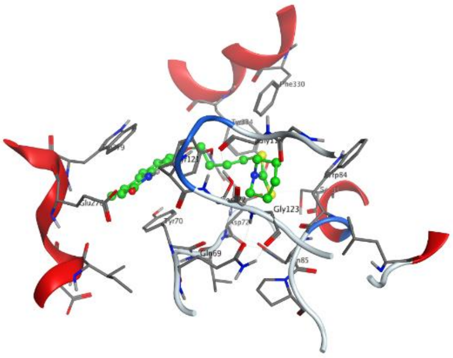
**9b**	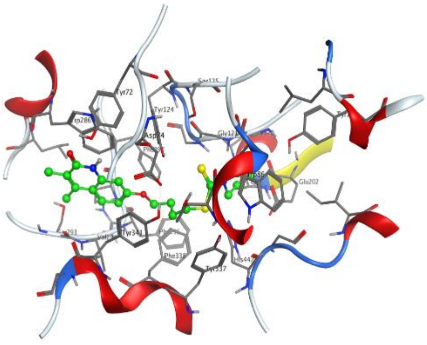	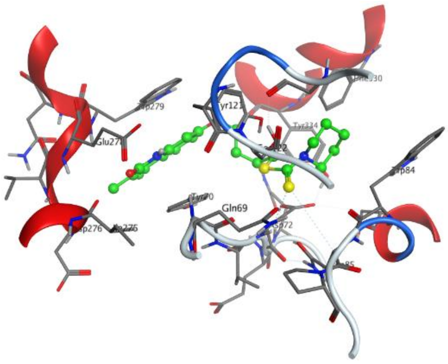

**donepezil**	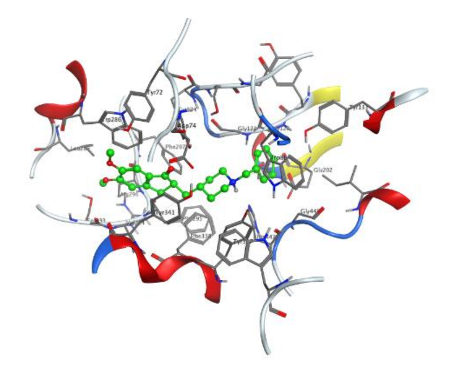	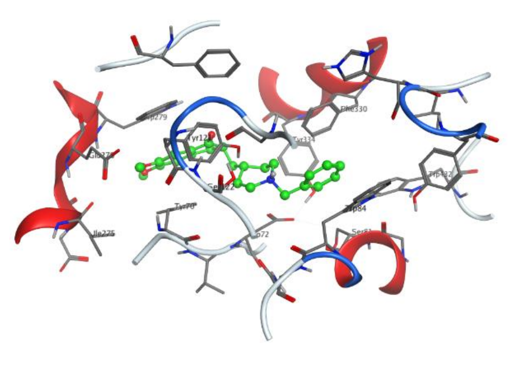
